# Synthetic Tyrosine tRNA Molecules with Noncanonical Secondary Structures

**DOI:** 10.3390/ijms20010092

**Published:** 2018-12-26

**Authors:** Kensaku Sakamoto, Akiko Hayashi

**Affiliations:** Laboratory for Nonnatural Amino Acid Technology, RIKEN Center for Biosystems Dynamics Research (BDR), 1-7-22 Suehiro-cho, Tsurumi, Yokohama 230-0045, Japan; akiko.matsumoto@riken.jp

**Keywords:** genetic code expansion, pyrrolysine tRNA, tertiary base pairs, tRNA secondary structure, amber suppression

## Abstract

The L-shape form of tRNA is maintained by tertiary interactions occurring in the core. Base changes in this domain can cause structural defects and impair tRNA activity. Here, we report on a method to safely engineer structural variations in this domain utilizing the noncanonical scaffold of tRNA^Pyl^. First, we constructed a naïve hybrid between archaeal tRNA^Pyl^ and tRNA^Tyr^, which consisted of the acceptor and T stems of tRNA^Tyr^ and the other parts of tRNA^Pyl^. This hybrid tRNA efficiently translated the UAG codon to 3-iodotyrosine in *Escherichia coli* cells, when paired with a variant of the archaeal tyrosyl-tRNA synthetase. The amber suppression efficiency was slightly lower than that of the “bench-mark” archaeal tRNA^Tyr^ suppressor assuming the canonical structure. After a series of modifications to this hybrid tRNA, we obtained two artificial types of tRNA^Tyr^: ZtRNA had an augmented D (auD) helix in a noncanonical form and the D and T loops bound by the standard tertiary base pairs, and YtRNA had a canonical auD helix and non-standard interloop interactions. It was then suggested that the ZtRNA scaffold could also support the glycylation and glutaminylation of tRNA. The synthetic diversity of tRNA would help create new tRNA–aminoacyl-tRNA synthetase pairs for reprogramming the genetic code.

## 1. Introduction

**Figure 1:**
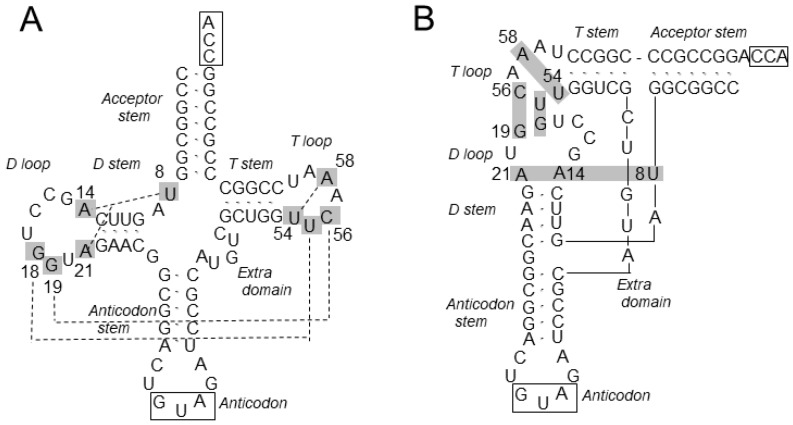
The cloverleaf (**A**) and L-shape (**B**) representations of the wild-type *M. jannaschii* tRNA^Tyr^, which belongs to class I tRNA. The nucleotides are all indicated in unmodified forms, and the invariant T54 and ψ55 are shown as U. The invariant nucleotides mentioned in the text are indicated in gray boxes, and the tertiary base pairs between them are represented by broken lines (**A**) or the extension of the gray boxes (**B**). Nucleotides are numbered by the standard system.

An L-shape form is the prominent structural feature of tRNA and is maintained through tertiary interactions taking place in the tRNA core, which encompasses the D arm, the T loop, and the intervening nucleotides between the four arms [1,2] (Figure 1). The D and T loops are bound together by the invariant G18-ψ55 and G19-C56 base pairs (“ψ” is pseudouridine, a modified U), while the D stem and its surrounding nucleotides are integrated into a structure called the augmented D (auD) helix [2]. A triple base pair between the invariant U8, A14, and A21 is formed as a part of this helix, while T54 is base-paired with A58 within the T loop. Most of the invariant nucleotides of the canonical tRNA are included in the core, indicating their importance in structure maintenance. An important effect of the tRNA folding into the L shape is to place the anticodon and 3’-terminal CCA sequence at the opposite extremities, with the correct distance and relative orientations between them. These parameters cannot greatly vary among different tRNA species, as the anticodon and CCA should fit into the designated places in the ribosome during translation. 

Despite this required structural uniformity, there is a significant variance in the tertiary interactions in the cores of naturally-occurring tRNAs [3,4,5]. The cytosolic tRNAs specific for the 20 amino acids (the canonical tRNAs) are categorized into two classes, and class II tRNAs (tRNA^Leu^, tRNA^Ser^, and the bacterial tRNA^Tyr^) have a long extra helix between the anticodon and T arms. Tertiary interactions in the auD helix are rearranged in class II tRNAs to support these helices, although many of the invariant nucleotides are common between the two categories, including the tertiary pairs indicated in Figure 1. The unique long helices of class II tRNAs, and a distinct core structure of tRNA^Cys^ as well, play a crucial role in the accurate aminoacylation by the cognate aminoacyl-tRNA synthetases (aaRSs) [6,7,8,9,10]. The noncanonical structure of pyrrolysine tRNA is also involved in the specific recognition by pyrrolysyl-tRNA synthetase [11,12], while the unique structure of selenocysteine tRNA is prerequisite for interacting with the cellular factors assisting the incorporation of this amino acid into certain enzymes [13]. Thus, the structural variations in the tRNA core contribute to the diversified amino-acid specificities of tRNAs, and consequently the variety of the genetically encoded amino acids.

**Figure 2:**
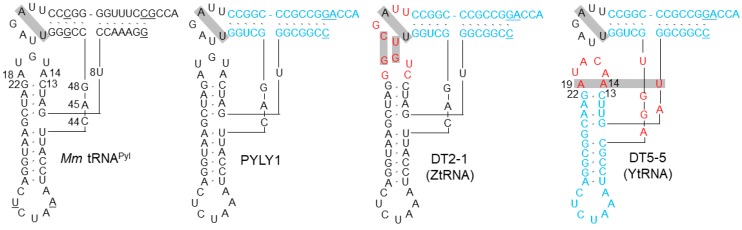
L-shape representations of *M. mazei* (*Mm*) tRNA^Pyl^, PYLY1 (the naïve hybrid between *Mm* tRNA^Pyl^ and *M. jannaschii* tRNA^Tyr^), and two representative variants isolated from selections. The nucleotides in blue and black are derived from *M. jannaschii* tRNA^Tyr^ and *Mm* tRNA^Pyl^, respectively. The nucleotides in red show randomized positions. The identity elements of tRNA^Pyl^ and tRNA^Tyr^ are underlined. A tertiary base pair in *Mm* tRNA^Pyl^ and presumed tertiary base pairs in the other tRNAs are indicated with grey boxes. Some nucleotides are numbered to highlight the difference between our numbering system and the standard one.

In the present study, we explored feasible modifications to the core structure with the purpose of creating the synthetic diversity of tRNA forms. We started with the tRNA^Pyl^ scaffold, as this tRNA has fewer invariant nucleotides than the canonical tRNA, seemingly with more changeability in the core′s nucleotides. The structural idiosyncrasies of tRNA^Pyl^ are concentrated in the core, such as a single or no bridging nucleotide between the acceptor and D stems, a small D loop, a six base-pair anticodon stem, and a three base extra domain (Figure 2). A crystallographic study showed that the elongated anticodon stem causes a shift of the entire D arm toward the T loop [11]. The lack of the invariant interloop base pairs, G18-ψ55 and G19-C56, also stands out. We found that the tRNA^Pyl^ scaffold supports in vivo aminoacylation by the archaeal tyrosyl-tRNA synthetase (TyrRS), although its cognate archaeal tRNA^Tyr^ originally assumes the canonical form of class I tRNA. Allowable sequence variations in the core were then explored by constructing a number of the libraries of tRNA variants with different randomized positions. The present study is the first report on the systematic and extensive engineering of the tRNA core structure. 

## 2. Results

### 2.1. Hybrid Molecules between tRNA^Pyl^ and tRNA^Tyr^

**Figure 3:**
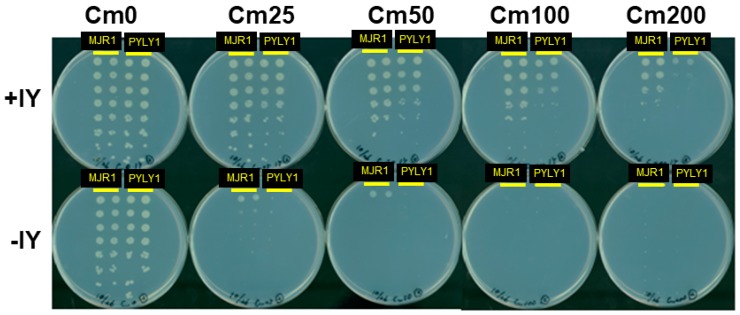
In vivo amber suppression activities of MJR1 and PYLY1. The cell suspensions of two *E. coli* clones transformed with each of the indicated variant genes were diluted successively (from top to bottom) and grown on the rich medium plates containing Cm at the indicated final concentrations with or without the supplementation of 3-iodotyrosine (IY).

Our numbering of the nucleotides in tRNA^Pyl^ is basically in accordance with the standard system. The missing nucleotides are assigned to positions 9, 46, and 47, while the D loop starts with position 14 and there is a numbering gap between the 3′ terminal position of this loop and position 22 in the D stem. Nucleotides in the anticodon stem, including the extra base pair, will not be mentioned throughout this study. The first tRNA construct was a naïve hybrid (PYLY1) between *Methanosarcina mazei* tRNA^Pyl^ and *Methanocaldococcus jannaschii* tRNA^Tyr^, comprising the acceptor and T stems of the tRNA^Tyr^ and the other parts from the tRNA^Pyl^ (Figure 2 and Table 1). PYLY1 was co-expressed in *Escherichia* coli cells, together with iodoTyrRS-*mj*, a variant of *M. jannaschii* TyrRS specific for 3-iodotyrosine [14] and a chloramphenicol acetyltransferase (CAT) gene with an in-frame UAG stop codon. A dose of 3-iodotyrosine was supplemented in the growth medium. PYLY1 was found to cause amber suppression depending on the presence of 3-iodotyrosine, indicating that it was recognized by the archaeal TyrRS (Figure 3). The amber suppression efficiency was slightly lower than that exhibited by the “bench-mark” *Mj* tRNA^Tyr^ amber suppressor, MJR1 [15]; PYLY1 conferred a resistance to chloramphenicol (Cm) at a concentration up to 100 μg/mL, while MJR1 conferred a resistance up to Cm200. 

The background activity was then compared between PYLY1 and the bench-mark MJR1. When *Mj* tRNA^Tyr^ was converted into an amber suppressor (MJR, Table 1), the resulting tRNA reportedly showed a low level of suppressor activity in the absence of *Mj* TyrRS in *E. coli* [16]. The change in the anticodon sequence probably elicited the recognition by endogenous aaRS. MJR1 has a few base substitutions that suppress this undesirable activity (Table 1) [15]. MJR1, when co-expressed with iodoTyrRS-*mj*, conferred marginal Cm resistance in the absence of 3-iodotyrosine, whereas such activity was hardly detected for PYLY1 (Figure S1A). PYLY1 is thus a poor substrate for the endogenous aaRSs, like MJR1. 

Instead of replacing the entire acceptor and T stems, the major identity determinants of the archaeal tRNA^Tyr^ (C1, G72 and A73) [17] were “transplanted” into *M. mazei* tRNA^Pyl^. The resulting variant PYLY2 (Table 1) was found to be as efficient an amber suppressor as PYLY1 in the presence of 3-iodotyrosine (Figure S1B). Consistently, the suppression ability of PYLY1 was abolished by eliminating the determinants, with the replacement of C1-G72 by GC/UA or A73 by G/U (Table 1 and Figure S1C). These findings showed that the archaeal TyrRS recognizes PYLY1 in the proper manner, depending on the presence of identity determinants.

Finally, other tRNA^Pyl^ sequences were examined if they are useful for aminoacylation by *Mj* TyrRS. The identity determinants of the archaeal tRNA^Tyr^ were transplanted into the bacterial tRNA^Pyl^ from *Desulfitobacterium hafniense* and the archaeal tRNA^Pyl^ from *Candidatus Methanomethylophilus* alvus, to create variants PYLYDh and PYLYCMa, respectively (Table 1). *Dh* tRNA^Pyl^ assumes a similar secondary structure to that of *Mm* tRNA^Pyl^, whereas *CM*a tRNA^Pyl^ has such idiosyncrasies as no nucleotide between the acceptor and D stems, a 4-nucleotide D loop, and an extra nucleotide in the 6-base pair anticodon stem [4]. Since the removal of this extra residue does not impair the in vivo tRNA activity [18], we used the sequence of this deletion variant of CMa tRNA^Pyl^ for the transplantation. PYLYCMa showed a higher suppression activity than PYLY1 and PYLY2, while PYLYDh was also aminoacylated by *Mj* TyrRS, although its suppressor activity was lower than that of PYLY1 (Table 1 and Figure S1B). These observations indicated that the tRNA^Pyl^ scaffold recognized by the archaeal TyrRS is not limited to the particular sequence of *Mm* tRNA^Pyl^.

### 2.2. Selections 1 and 2: Sequence Variations in the auD Helix

The tRNA core is divided into two parts: The D and T loops bound with each other, and the auD helix, which comprises the D stem, the proximal nucleotides of the D loop, and the intervening nucleotides between the four arms [2]. We first explored allowable variations in base sequence in the auD helix, by randomizing twelve nucleotides at positions 8, 11–13, 22–26, 44, 45, and 48 of PYLY1 (Table S1). The cells transformed with a resulting library were selected for Cm resistance, and plasmids were then extracted from the cells exhibiting a resistance to Cm at a final concentration of 25 μg/mL. These plasmids were then introduced into *E. coli* cells again to confirm the Cm resistance. The PYLY1 variants thus isolated are listed in Table S1. Five of the isolated sequences had intact D stems with various sequences. We represent an intact D stem by its base sequence from position 10 to position 13. For instance, the GGCT1 variant has a GGCU stem. The five isolated variants conferred a resistance against Cm25–Cm50 (Figure S2), which were lower levels than that achieved by the parent PYLY1. 

To investigate the correlation between D-stem sequence and the surrounding nucleotides in the auD helix, we randomized positions 8, 26, 44, 45, and 48 in PYLY1 with different D stems, such as the GGCU and GCCC stems, and the D stem (GUUC) of *Mj* tRNA^Tyr^ (Libraries 2a–2c, respectively; Table S1). The selection was conducted with a more stringency than Selection 1; we kept only the variants conferring a resistance against Cm100 or more. Isolated clones were re-cloned and the ability to confer Cm resistance was thus confirmed, to exclude the contribution of any spontaneous mutation outside the tRNA gene in the vector plasmid. Sixteen tRNA sequences isolated from the selection are listed in Table 2. These tRNAs, except for one variant, exhibited a resistance against Cm100 (Figure S3), a level comparable with that of PYLY1 and higher than those of the parent GGCT1 and GCCC1 variants. Preferred bases at the randomized positions varied among the tested D-stem sequences, and were found to be R26, G44, U45, and Y48 for the GGCU stem (“R” and “Y” represent purines and pyrimidines, respectively); U8, R26, K44, G45, and A48 for GCCC (“K” represents G or U, respectively); and U8, R26, W44, G45, and A48 for GUUC (“W” represents A or U). Although the number of samples is small, these different preferences possibly reflect the structural relationship between the D stem and the surrounding nucleotides in the auD helix. 

### 2.3. Selection 3: Sequence Variation in the D Loop against the T Loop of Mm tRNA^Pyl^

Next, we randomized nucleotides in the 5-base D loop, the 3-base extra domain, and at position 8 in PYLY1, while at the same time the sequences of the T loop and D stem, both derived from *Mm* tRNA^Pyl^, were not changed. All five of the tRNA sequences (DLE1–DLE5) isolated from this library (Library 3) are listed in Table 3. Their suppressor activities in terms of the Cm resistance were found to be comparable with or higher than that of the parent PYLY1; DLE4 almost matched the bench-mark *Mj* tRNA^Tyr^ suppressor in the activity (Table 3 and Figure S4). All of the isolated variants had a UGU sequence in the middle of the D loop, while the same sequence occurs in at the corresponding positions in the parent PYLY1/*Mm* tRNA^Pyl^ (Figure 2). Since the tRNA variants of Library 3 shared the base sequence of the T loop with the parent tRNAs, the noncanonical interloop interactions in the tRNA^Pyl^ were probably replicated in the isolated variants. A difference is that the nucleotides at positions 14 and 18 presumably formed a Watson-Crick base pair in all of the isolated variants, whereas the bases at the corresponding positions (G14 and A18) of *Dh* tRNA^Pyl^ face away from each other in the reported crystal structure [11]. 

### 2.4. Selection 4: Sequence Covariation between the D and T Loops in PYLY1 and GGCT1 (ZtRNA)

To investigate covariation between the D- and T-loop sequences, we simultaneously randomized the nucleotides in them for two different auD helices. The tRNA^Pyl^ and canonical tRNA scaffolds include the same number of nucleotides in the T loop, and the invariant U54-A58 base pair also exists in tRNA^Pyl^. These two positions were not randomized in Libraries 4a and 4b, based on PYLY1 and GGCT1, respectively. Nine different tRNA sequences (DT2-1–DT2-4 and DT3-1–DT3-5) were isolated from these two libraries (Table 4). The amber suppressor activity, ranging from Cm50 to Cm200, was confirmed by re-cloning, and three variants matched the bench-mark *Mj* tRNA^Tyr^ suppressor in the activity (Figure S5). All nine of the isolated tRNAs had G16, G17, U55, C56, and R57 in common. These nucleotides also occur in the bovine mitochondria tRNA^Ser^_UGA_ [19,20], which shares the tRNA^Pyl^ scaffold. It has been proposed that G16-U55 are base-paired with G17-C56, just like between G18-ψ55 and G19-C56 in the canonical tRNA [20,21]. We name this particular type of the tRNA^Pyl^ scaffold as ZtRNA (Figure 2), which is characterized by the presence of a G_16_G_17_/U_54_U_55_C_56_R_57_A_58_ sequence. We noticed that none of the variants isolated from Selection 3 and 4 had the same D- and T-loop sequences as those of the parent tRNA^Pyl^. This discrepancy was probably a result of the optimization in the core structure in favor of the interaction with the archaeal TyrRS.

### 2.5. Selection 5: Selection of Structural Hybrids having the Canonical auD Helix and Noncanonical Interloop Interactions (YtRNA)

ZtRNA is a structural hybrid having a noncanonical auD helix and the canonical interactions between the D and T loops. We then explored the feasibility of another hybrid with the reversed roles for canonical and noncanonical structures. Three variant libraries (DT4, DT5, and DT6) with different D-loop sizes (five, six, and seven nucleotides, respectively) were designed, based on the sequence of *Mj* tRNA^Ty^^r^. The T-loop sequence of this tRNA was changed to that of *Mm* tRNA^Pyl^, while randomizing nucleotides at positions 8 and 9 as well as those in the D loop and the extra domain. The length of this domain was shortened from five to four; a four-base extra domain is found in *E. coli* tRNA^Glu^. Two suppressors were isolated each from the DT4 and DT6 libraries, conferring the resistance against Cm25–Cm100 in the presence of 3-iodotyrosine (Table 5 and Figure S6). Eleven suppressors were isolated from the DT5 library, conferring the resistance against Cm50–Cm200 in the presence of the tyrosine derivative. The DT5-5 variant matched the bench-mark tRNA^Tyr^ suppressor in the activity. Sequence determination revealed that U8, A14, and A at the last D-loop position occur simultaneously in the sequences of eight DT5 variants, including DT5-5, and the two DT6 variants. These nucleotides apparently correspond to the invariant U8, A14, and A21 of the canonical tRNA, which form a triple base pair in the auD helix. On the other hand, the interactions between the D and T loop were noncanonical in these DT5 and DT6 variants, with the lack of the G_16_G_17_/U_54_U_55_C_56_R_57_A_58_ motif. We name this new scaffold YtRNA (Figure 2), which is a particular type of class I tRNA characterized by the canonical auD helix with the U_8_A_14_A_21_ motif and noncanonical interloop interactions. 

### 2.6. Selection 6: ZtRNA Scaffold Possibly Supports Glycylation and Glutaminylation in E. coli

Finally, we examined if the tRNA^Pyl^ scaffold is compatible with the acylation with other amino acids than tyrosine and pyrrolysine. Two variant libraries were designed, based on GGCT1, a variant of PYLY1 (Table S1). The C1-G72 pair was replaced with G1-C72, and in addition, A73 was randomly replaced with other nucleotides in Library 6a. C1, G72, and A73 were each randomly replaced with other nucleotides in Library 6b. Additionally, the ten positions in the D and T loops, other than the U54-A58 pair, were randomized in both libraries. Selection was conducted with a low stringency, and four variants were then isolated from Library 6a. These variants (GLY1–GLY4) conferred a resistance against Cm5–15 in the absence of 3-iodotyrosine, and the supplementation of this amino acid did not increase the level of the Cm resistance (Table 6 and Figure S7A). This observation suggested that these variants were aminoacylated by endogenous aaRS. Sequence determination showed that all variants (GLY1–GLY4) had an intact G_16_G_17_/U_54_U_55_C_56_R_57_A_58_ motif and U73 in common. One of the major identity determinants of tRNA^Gly^ is U73 [22] and an amber suppressor tRNA derived from tRNA^Gly^ reportedly incorporates glycine at UAG [23,24]. Based on the presence of U73 in the isolated variants, we inferred that they might be aminoacylated by the endogenous glycyl-tRNA synthetase (GlyRS). On the other hand, five false positive clones (FP1–FP5), excluded at a verification process, were found to lack the G_16_G_17_/U_54_U_55_C_56_R_57_A_58_ motif (Table S1), which suggests that the ZtRNA format was important for the suppressor activities of GLY1–GLY4.

Two variants (GLN1 and GLN2) were isolated from Library 6b; both had U1 forming a mismatched pair at the end of the acceptor stem, the discriminator base G73, and an intact G_16_G_17_/U_54_U_55_C_56_R_57_ motif (Table 6). These characteristics were not shared by false positive clones (FP6 and FP7) (Table S1). GLN1 and GLN2 exhibited suppressor activities ranging from Cm5 to Cm15 regardless of the presence of 3-iodotyrosine, like GLY1–GLY4 (Table 6 and Figure S7B). The tRNA recognition by *E. coli* glutaminyl-tRNA synthetase (GlnRS) involves U1-A73 (or a weak base pair between these positions), C2-G72, U35, and G73 [25,26,27,28,29]. Then, we changed the first two base pairs in the acceptor stem of GLN1 to mimic the corresponding part of *E. coli* tRNA^Gln^. The resulting variant, GLNa (Table 6), showed a slightly higher efficiency in the amber suppression (Figure S7C). These findings suggested that these ZtRNA variants might be recognized by *E. coli* GlnRS. Further experiments to purify and analyze the products synthesized based on the poor suppressor activities of the GLN and GLY variants will be necessary to confirm the identities of the amino acids incorporated at UAG. 

## 3. Discussion

In the present study, we showed that various noncanonical secondary structures of tRNA can support aminoacylation by the archaeal TyrRS, although the natural substrate, *Mj* tRNA^Tyr^, is a class I tRNA with the canonical form. The crystal structure of *Mj* tRNA^Tyr^ complexed with *Mj* TyrRS has been reported to show that the synthetase binds to the tRNA in the anticodon moiety and the distal end of the acceptor stem, with no direct contact in the core [30]. Although TyrRS is a class I synthetase, the archaeal and bacterial TyrRS species bind to the major groove side of the acceptor stem [30,31], like PylRS [11]. The absence of an extensive contact between *Mj* TyrRS and the core of the cognate tRNA^Tyr^ probably allowed the tRNA^Tyr^ to assume various forms with no serious damaged to the in vivo activity. When we are focused on individual forms, we notice that only specific sequences supported the activity, and surmise that the core structure was thus tuned to place the anticodon and CCA end, the two contact sites, in right positions relative to each other in space. This claim would be tested by structure determination or simulation to reveal the effect of a specific core sequence on dynamics in the overall structure of tRNA. 

The method that we developed for creating structural variations in the tRNA core involves the division of this domain into separate parts to be modified independently. Thus, the nucleotide sequence in the auD-helix was optimized separately from the other parts of the core in Selections 1 and 2. The D-loop sequence was randomized while fixing the T-loop sequence to be that of *Mm* tRNA^Pyl^ in Selections 3 and 5. The sequences in these two loops were changed simultaneously in Selection 4. Thus, we found that four different scaffolds—the canonical tRNA, tRNA^Pyl^, ZtRNA, and YtRNA—are aminoacylated by *Mj* TyrRS tRNA with similar in vivo efficiencies.

ZtRNA is a particular class of the tRNA^Pyl^ format presumably with the canonical interloop interactions involving the G_16_G_17_/U_54_U_55_C_56_R_57_A_58_ motif. On the other hand, YtRNA is regarded as a variation of the canonical tRNA that has a smaller D loop and lacks the standard interloop interactions. It was suggested that ZtRNA can be recognized as a substrate by *E. coli* GlyRS and GlnRS. There have been reports on the misaminoacylation of tRNA^Pyl^ by aaRSs that normally acylate canonical tRNAs [32,33,34,35]. These findings imply that the core structure could be changed also in tRNA species other than the archaeal tRNA^Tyr^, while their interactions with cognate aaRSs are maintained at the same time.

Transfer tRNA interacts with multiple factors (modification enzymes, aaRS, EF-Tu, ribosome, etc.) to become mature and play a pivotal role in translation, while its structure is maintained through the complex network of tertiary interactions. These conditions pose an engineering challenge when synthetic tRNAs are developed [13,36]. The tRNA core has been involved in recent structural manipulations of the molecule for genetic code expansion. Pyrrolysine tRNA was thus modified in the extra domain to create mutually orthogonal variant pairs [37,38], and in the D and T loops to increase in vivo translation efficiency [39]. The manipulation for rewiring of tRNA^Sec^ to the amber UAG codon involved a part of the auD helix [40]. In addition, the replacement of the original five-base extra domain by a selected four-base sequence reportedly improved the in vitro affinity of *E. coli* tRNA^Gln^ for the cognate synthetase [41]. We noted that tRNA^Pyl^ lacks the conserved nucleotides to bind the D and T loops together, and previous melting experiments indicated that these loops of tRNA^Pyl^ “open” at a lower temperature than those of tRNA^Lys^ [32]. The implied weak binding between the loops might loosen constraints in nucleotide sequence. *Mm* tRNA^Pyl^ includes 25 nucleotides in the core region, and the number of the possible permutations reaches 1.1 × 10^15^. Although the possibilities are thus enormous in theory, how many distinct and functional structures are represented among them is an important question related to the development of new tRNA-aaRS pairs.

Since tRNA-aaRS pairs match amino acids with codons, more pairs will be necessary when the degeneracy of codons is reduced and multiple codons are available for specifying synthetic amino acids [42]. A straightforward approach of developing new pairs is to replace identity elements in a tRNA and then engineer aaRS variants adapted to this change. This approach requires a remodeling of the binding pockets for identity nucleotides in aaRS, and has been successful in only a few cases [43,44]. We propose another path to change the tRNA specificity of aaRS. The L-shape form of tRNA is the structural framework in which the positions of identity nucleotides are recognized by aaRS. The determinants might not be recognized, or recognized in a different way, by aaRS in a distorted framework, if they are intact. For example, it was reported that the removal of residue 26, a part of the auD helix, from *E. coli* tRNA^Arg^ shifted the major identity position from position 35 to 34, and allowed the amber suppression by this tRNA [45,46]. A change in the structural framework would provide a new opportunity for developing aaRS variants. Thus, we could take advantage of synthetic variations in the structure of the core, a crucial part for maintaining the framework, with the purpose of controlling the tRNA specificity of aaRS.

## 4. Materials and Methods

### 4.1. Amino Acid and E. coli Strains

3-Iodotyrosine was purchased from Watanabe Chemical Industries (Hiroshima, Japan). HB101 and MV1184 were purchased from Takara BIO Inc. (Shiga, Japan).

### 4.2. tRNA Genes

All tRNA genes, including the genes with randomized positions, were each constructed by annealing three DNA oligonucleotides: R1 and R2 oligomers and a longer oligomer with the prefix “F-” (Table S2). The F-oligomers each contain a whole tRNA sequence with certain sequences at the ends, to be ligated into the *Bst*XI site of the pTYR2541 plasmid. The R1- and R2-oligomers anneal with the 3′- and 5′-ends, respectively, of the F-oligomer, and the annealed product thus has a gap in one strand, which was filled up in *E. coli* cells. The three oligomers were phosphorylated at the 5′-end prior to annealing. All oligomers were commercially synthesized by Eurofin Genomics Co., Ltd. (Tokyo, Japan). The sequences of the constructed tRNA genes were confirmed by sequence analysis.

### 4.3. Plasmids

The plasmid pTYR2541 (Figure S8) carries the gene coding for iodoTyrRS-*mj* [14] with an Asp286Arg substitution [30] under the control of the *tyrS* promoter; a tRNA gene at the *Bst*XI site under the control of the *lpp* promoter and *rrnC* terminator [23,24]; a CAT mutant gene with UAG substituting for a Thr codon at position 10; and a kanamycin resistance gene from plasmid pHSG299 (Takara BIO). This plasmid was based on the cloning vector pACYC184 [GenBank X06403.1], from which we removed a 58-base sequence, ^1990^GCGCTTG…TGGGCGC^2047^, complementary with the sequence at positions 3365–3422. The aforementioned elements were then incorporated into the modified vector.

### 4.4. In Vivo tRNA Activity Assay

The amber suppressor activity of any tRNA with 3-iodotyrosine was examined by transforming the *E. coli* MV1184 cells with the pTYR2541 plasmid carrying this tRNA and then growing the transformants on a rich media plate containing Cm at the indicated final concentration and 3-iodotyrosine at a final concentration of 0.1 mM at 37 °C. 

### 4.5. Isolation of tRNA Variants

*E. coli* HB101 cells were transformed with a library of tRNA variants carried by the pTYR2541 plasmid, and then the library plasmids were extracted from the transformants. The library size was 10^5^–10^6^ at this stage. The MV1184 cells were then transformed with the extracted library plasmids for selection based on Cm resistance. The Cm-resistant clones were kept, and the plasmids extracted from them were introduced into the host cells again, to confirm the ability to confer Cm resistance and eliminate false positive clones. A part of the CAT gene encompassing position 10 was sequenced for each of the isolated positive plasmids, to confirm that the resistance was not caused by a reversion from UAG to a sense codon. When indicated, tRNA genes from the isolated plasmids were re-cloned in pTYR2541 and verified for amber suppression activity. 

## Figures and Tables

**Table 1:** In vivo suppressor activities with 3-iodotyrosine and base sequences of *M. jannaschii* tRNA^Tyr^ suppressors, *M. mazei* tRNA^Pyl^, and the hybrid tRNAs between *Mj* tRNA^Tyr^ and tRNA^Pyl^ molecules.

Variant	Amber Suppression	Sequence ^1^
MJR	Cm200	CCGGCGGUAGUUCAGCCUGGUAGAACGGCGGACUCUAAAUCCGCAUGUCGCUGGUUCAAAUCCGGCCCGCCGGACCA
MJR1	Cm200	CCGGCGGUAGUUCAGCAGGGCAGAACGGCGGACUCUAAAUCCGCAUGGCGCUGGUUCAAAUCCGGCCCGCCGGACCA
*Mm* tRNA^Pyl^	N. D.	GGAAACCUGAUCAUGUAGAUCGAAUGGACUCUAAAUCCGUUCAGCCGGGUUAGAUUCCCGGGGUUUCCGCCA
PYLY1	Cm100	CCGGCGGUGAUCAUGUAGAUCGAAUGGACUCUAAAUCCGUUCAGGCUGGUUAGAUUCCGGCCCGCCGGACCA
PYLY2	Cm100	CGAAACCUGAUCAUGUAGAUCGAAUGGACUCUAAAUCCGUUCAGCCGGGUUAGAUUCCCGGGGUUUCGACCA
PYLY1 (G1C72)	< Cm25	GCGGCGGUGAUCAUGUAGAUCGAAUGGACUCUAAAUCCGUUCAGGCUGGUUAGAUUCCGGCCCGCCGGACACCA
PYLY1 (U1A72)	< Cm25	UCGGCGGUGAUCAUGUAGAUCGAAUGGACUCUAAAUCCGUUCAGGCUGGUUAGAUUCCGGCCCGCCGGAAACCA
PYLY1 (G73)	<Cm25	CCGGCGGUGAUCAUGUAGAUCGAAUGGACUCUAAAUCCGUUCAGGCUGGUUAGAUUCCGGCCCGCCGGGCCA
PYLY1 (U73)	<Cm25	CCGGCGGUGAUCAUGUAGAUCGAAUGGACUCUAAAUCCGUUCAGGCUGGUUAGAUUCCGGCCCGCCGGUCCA
PYLY Dh	Cm50	CGGGGGUGGAUCGAAUAGAUCACACGGACUCUAAAUUCGUGCAGGCGGGUGAAACUCCCGUACUCCCGACCA
PYLY CMa	Cm200	CGGGGACGGUCCGGCGACCAGCGGGUCUCUAAAACCUGCCAGCGGGGUUCGACGCCCCGGUCUCUGACCA

^1^ The D, T, and anticodon stems are underlined. The bases in blue and black letters are derived from *M. jannaschii* tRNA^Tyr^ and tRNA^Pyl^, respectively, when they coexist in a sequence. The base substitutions in MJR1 described in ref. 15 and the designed substitutions in the PYLY1 variants are shown in bold green letters.

**Table 2:** In vivo suppressor activities with 3-iodotyrosine and base sequences in the cores of the PYLY1 variants obtained from Selection 2.

Variant	Amber Suppression	Sequence in the Core ^1^
GGCT2	Cm100	^8^CGGCUAUGUAAGCCA^26^…^44^GUCGCUGGUUAGAUUCCGGC^65^
GGCT3	Cm100	^8^AGGCUAUGUAAGCCG^26^…^44^GUUGCUGGUUAGAUUCCGGC^65^
GGCT4	Cm100	^8^AGGCUAUGUAAGCCA^26^…^44^GUUGCUGGUUAGAUUCCGGC^65^
GGCT5	Cm50	^8^UGGCUAUGUAAGCCG^26^…^44^GUCGCUGGUUAGAUUCCGGC^65^
GCCC2	Cm100	^8^UGCCCAUGUAGGGCG^26^…^44^UGAGCUGGUUAGAUUCCGGC^65^
GCCC3	Cm100	^8^UGCCCAUGUAGGGCA^26^…^44^GGAGCUGGUUAGAUUCCGGC^65^
GCCC4	Cm100	^8^UGCCCAUGUAGGGCG^26^…^44^UGUGCUGGUUAGAUUCCGGC^65^
GCCC5	Cm100	^8^UGCCCAUGUAGGGCG^26^…^44^GGAGCUGGUUAGAUUCCGGC^65^
GTTC1	Cm100	^8^UGUUCAUGUAGAACG^26^…^44^UGAGCUGGUUAGAUUCCGGC^65^
GTTC2	Cm100	^8^UGUUCAUGUAGAACA^26^…^44^AGAGCUGGUUAGAUUCCGGC^65^
GTTC3	Cm100	^8^UGUUCAUGUAGAACA^26^…^44^CGAGCUGGUUAGAUUCCGGC^65^
GTTC4	Cm100	^8^AGUUCAUGUAGAACG^26^…^44^UGAGCUGGUUAGAUUCCGGC^65^
GTTC5	Cm100	^8^UGUUCAUGUAGAACG^26^…^44^AGAGCUGGUUAGAUUCCGGC^65^
GTTC6	Cm100	^8^UGUUCAUGUAGAACG^26^…^44^UGCGCUGGUUAGAUUCCGGC^65^
GTTC7	Cm100	^8^UGUUCAUGUAGAACA^26^…^44^UGAGCUGGUUAGAUUCCGGC^65^
GTTC8	Cm100	^8^UGUUCAUGUAGAACG^26^…^44^UGUGCUGGUUAGAUUCCGGC^65^

^1^ The sequences from positions 8–26 and positions 44–65 are indicated. The complete sequences of the variants are shown in Table S1. The D and T stems are underlined. The bases in red and green letters show randomized positions and designed base substitutions, respectively. The bases in blue and black letters are derived from *M. jannaschii* tRNA^Tyr^ and *M. mazei* tRNA^Pyl^, respectively. Frequently occurring bases are shown in bold letters.

**Table 3:** In vivo suppressor activities with 3-iodotyrosine and base sequences in the cores of the PYLY1 variants obtained from Selection 3.

Variant	Amber Suppression	Sequence in the Core ^1^
DLE1	Cm100	^8^UGAUCU**UGU**AGAUCG^26^…^44^UGAGCUGGUUAGAUUCCGGC^65^
DLE2	Cm100	^8^AGAUCA**UGU**UGAUCA^26^…^44^AGCGCUGGUUAGAUUCCGGC^65^
DLE3	Cm100	^8^UGAUCG**UGU**CGAUCG^26^…^44^AGAGCUGGUUAGAUUCCGGC^65^
DLE4	Cm200	^8^AGAUCC**UGU**GGAUCG^26^…^44^CUGGCUGGUUAGAUUCCGGC^65^
DLE5	Cm100	^8^UGAUCA**UGU**UGAUCC^26^…^44^UGCGCUGGUUAGAUUCCGGC^65^

^1^ The sequences from positions 8–26 and positions 44–65 are indicated. The complete sequences of the variants are shown in Table S1. The D and T stems are underlined. The bases in red letters show randomized positions. The bases in blue and black letters are derived from *M. jannaschii* tRNA^Tyr^ and *M. mazei* tRNA^Pyl^, respectively. The consensus UGU sequence is shown in bold letters.

**Table 4:** In vivo suppressor activities with 3-iodotyrosine and base sequences in the cores of the PYLY1 and GGCT1 variants obtained from Selection 4.

Variant	Amber Suppression	Sequence in the Core ^1^
DT2-1	Cm200	^8^UGAUCCU**GG**GGAUCG^26^…^44^CAGGCUGGUUCGAUUCCGGC^65^
DT2-2	Cm100	^8^UGAUCAA**GG**UGAUCG^26^…^44^CAGGCUGGUUCAAUUCCGGC^65^
DT2-3	Cm100	^8^UGAUCAU**GG**UGAUCG^26^…^44^CAGGCUGGUUCAACUCCGGC^65^
DT2-4	Cm50	^8^UGAUCAA**GG**AGAUCG^26^…^44^CAGGCUGGUUCGAUCCCGGC^65^
DT3-1	Cm200	^8^UGGCUCU**GG**GAGCCA^26^…^44^CUCGCUGGUUCGACUCCGGC^65^
DT3-2	Cm200	^8^UGGCUAC**GG**UAGCCA^26^…^44^CUCGCUGGUUCGACCCCGGC^65^
DT3-3	Cm100	^8^UGGCUCU**GG**UAGCCA^26^…^44^CUCGCUGGUUCGAUUCCGGC^65^
DT3-4	Cm50	^8^UGGCUCG**GG**GAGCCA^26^…^44^CUCGCUGGUUCGAGUCCGGC^65^
DT3-5	Cm100	^8^UGGCUUA**GG**GAGCCA^26^…^44^CUCGCUGGUUCGAUUCCGGC^65^

^1^ The sequences from positions 8–26 and positions 44–65 are indicated. The complete sequences of the variants are shown in Table S1. The D and T stems are underlined. The bases in red and green letters show randomized positions and designed base substitutions, respectively. The bases in blue and black letters are derived from *M. jannaschii* tRNA^Tyr^ and *M. mazei* tRNA^Pyl^, respectively. The consensus G16, G17, U55, C56, and R57 are shown in bold letters.

**Table 5:** In vivo suppressor activities with 3-iodotyrosine and base sequences in the cores of the variants of *M. jannaschii* tRNA^Tyr^ with the T-loop sequence of *M. mazei* tRNA^Pyl^ obtained from Selection 5.

Variant	Amber Suppression	Sequence in the Core ^1^
DT4-1	Cm25	^8^AAGUUCAAGGUGAACG^26^…^44^AGGUGCUGGUUAGAUUCCGGC^5^
DT4-2	Cm50	^8^UAGUUCGUACUGAACG^26^…^44^AGUUGCUGGUUAGAUUCCGGC^65^
DT5-1	Cm100	^8^**U**AGUUC**A**ACAA**A**GAACG^26^…^44^AAAUGCUGGUUAGAUUCCGGC^65^
DT5-2	Cm50	^8^**U**AGUUC**A**AAGGGGAACG^26^…^44^AAGUGCUGGUUAGAUUCCGGC^65^
DT5-3	Cm100	^8^**U**GGUUC**A**AAGUCGAACG^26^…^44^AGGAGCUGGUUAGAUUCCGGC^65^
DT5-4	Cm100	^8^**U**AGUUC**A**AACA**A**GAACG^26^…^44^UGGUGCUGGUUAGAUUCCGGC^65^
DT5-5	Cm200	^8^**U**AGUUC**A**ACAU**A**GAACG^26^…^44^AGGUGCUGGUUAGAUUCCGGC^65^
DT5-6	Cm100	^8^**U**AGUUC**A**UAGU**A**GAACG^26^…^44^AGGUGCUGGUUAGAUUCCGGC^65^
DT5-7	Cm100	^8^**U**AGUUC**A**UGUC**A**GAACG^26^…^44^AAGAGCUGGUUAGAUUCCGGC^65^
DT5-8	Cm100	^8^AAGUUC**A**ACGCGGAACG^26^…^44^AGGAGCUGGUUAGAUUCCGGC^65^
DT5-9	Cm100	^8^**U**AGUUC**A**AUGG**A**GAACG^26^…^44^CUGUGCUGGUUAGAUUCCGGC^65^
DT5-10	Cm100	^8^**U**GGUUC**A**GAGU**A**GAACG^26^…^44^AGAUGCUGGUUAGAUUCCGGC^65^
DT5-11	Cm100	^8^**U**GGUUC**A**AUUA**A**GAACG^26^…^44^AGGUGCUGGUUAGAUUCCGGC^65^
DT6-1	Cm100	^8^**U**AGUUC**A**CUUGU**A**GAACG^26^…^44^AGGAGCUGGUUAGAUUCCGGC^65^
DT6-2	Cm100	^8^**U**AGUUC**A**ACUUU**A**GAACG^26^…^44^GAAUGCUGGUUAGAUUCCGGC^65^

^1^ The sequences from positions 8–26 and positions 44–65 are indicated. The complete sequences of the variants are shown in Table S1. The D and T stems are underlined. The bases in red letters show randomized positions. The bases in blue and black letters are derived from *M. jannaschii* tRNA^Tyr^ and tRNA^Pyl^, respectively. The consensus U8, A14, and A at the last position of the D-loop are shown in bold letters.

**Table 6:** In vivo suppressor activities and base sequences of the GGCT1/PYLY1 variants obtained from Selection 6.

Variant	Amber Suppression ^1^	Sequence ^2^
GLY1	Cm15	GCGGCGGUGGCUGU**GG**UAGCCAAAUGGACUCUAAAUCCGUUCUCGCUGGUUCGAGUCCGGCCCGCCGGACUCCA
GLY2	Cm5	GCGGCGGUGGCUGG**GG**UAGCCAAAUGGACUCUAAAUCCGUUCUCGCUGG UUCGAUUCCGGCCCGCCGGACUCCA
GLY3	Cm5	GCGGCGGUGGCUAU**GG**UAGCCAAAUGGACUCUAAAUCCGUUCUCGCUGG UUCGAGCCCGGCCCGCCGGACUCCA
GLY4	Cm5	GCGGCGGUGGCUCA**GG**AAGCCAAAUGGACUCUAAAUCCGUUCUCGCUGG UUCGAUUCCGGCCCGCCGGACUCCA
GLN1	Cm15	UCGGCGGUGGCUAU**GG**UAGCCAAAUGGACUCUAAAUCCGUUCUCGCUGG UUCGACUCCGGCCCGCCGGAC**G**CCA
GLN2	Cm5	UCGGCGGUGGCUCU**GG**GAGCCAAAUGGACUCUAAAUCCGUUCUCGCUGG UUCGACUCCGGCCCGCCGGAU**G**CCA
GLNa	Cm15—25	UGGGCGGUGGCUAUGGUAGCCAAAUGGACUCUAAAUCCGUUCUCGCUGG UUCGACUCCGGCCCGCCCAGCCA

^1^ The amber suppression occurred in both the presence and absence of 3-iodotyrosine at the indicated levels. ^2^ The D, T, and anticodon stems are underlined. The bases in blue and black letters are derived from *M. jannaschii* tRNA^Tyr^ and *M. mazei* tRNA^Pyl^, respectively. The bases in red and green letters show randomized positions and designed base substitutions, respectively. Bases in bold red letters are the structural features mentioned in the text.

## Supplementary Materials

Supplementary materials can be found at http://www.mdpi.com/1422-0067/20/1/92/s1.

## Abbreviations

auD	Augmented D
aaRS	Aminoacyl-tRNA synthetase
TyrRS	Tyrosyl-tRNA synthetase
GlyRS	Glycyl-tRNA synthetase
GlnRS	Glutaminyl-tRNA synthetase
