# Supplementary material for: Synthetic Tyrosine tRNA Molecules with Noncanonical Secondary Structures
Source: Int J Mol Sci. 2018 Dec 26;20(1):92. doi: 10.3390/ijms20010092 (PMC6337575; doi:10.3390/ijms20010092)
Supplement: Supplementary file 1 [file ijms-20-00092-s001.zip › Supplementary materials/Supplemantary Figures S1 to S8 revised.docx]

**Supplementary Materials**

**Figures 1—7**

**A.**


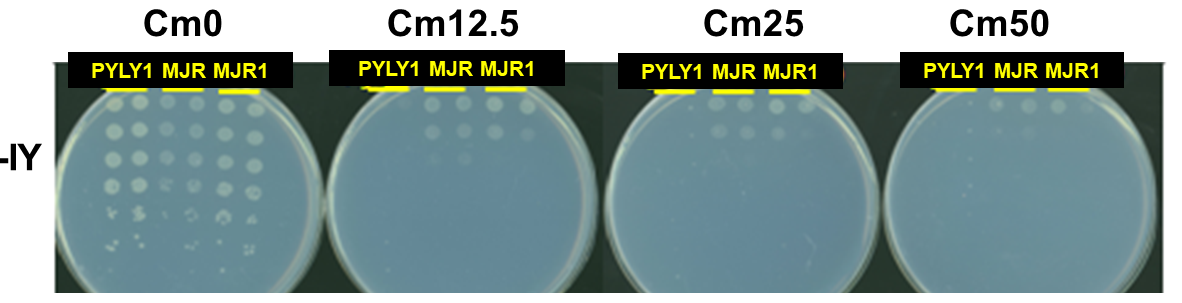


**B.**


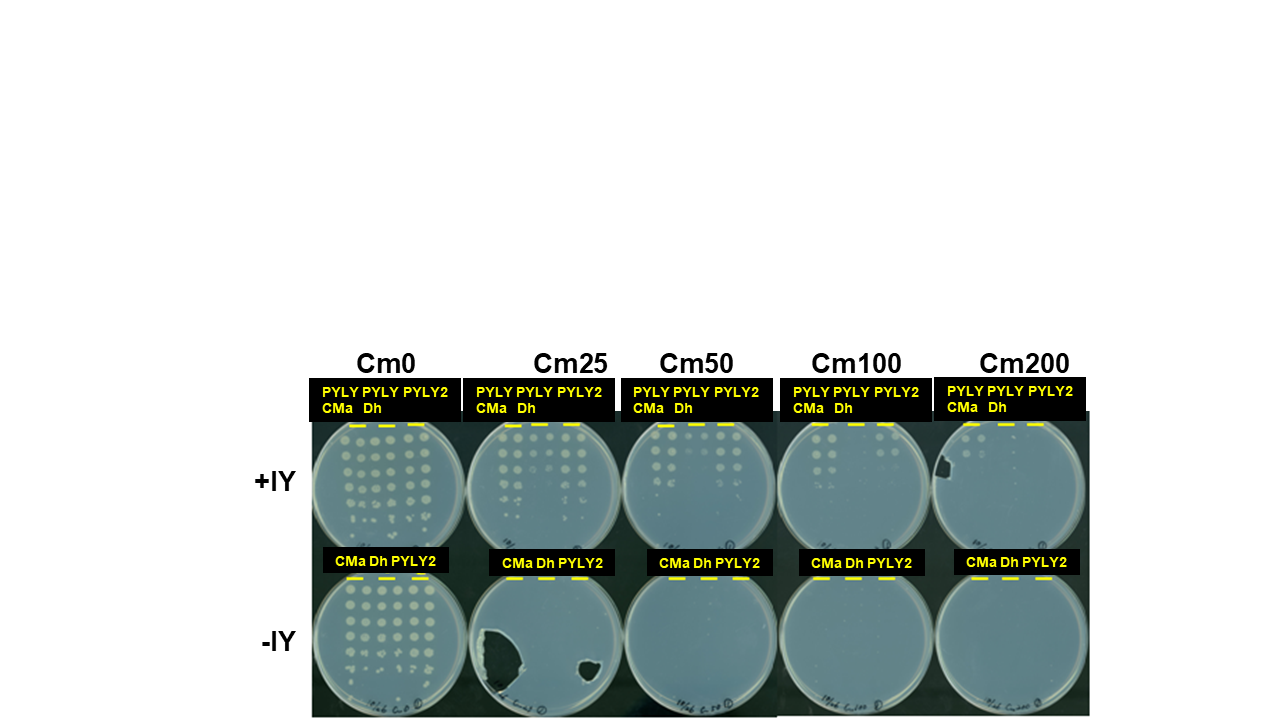


**Figure S1.** Amber suppressor activities of *Mj* tRNA^Tyr^ molecules and the designed variants with the tRNA^Pyl^ scaffold. (**A**) The background activities of MJR, MJR1, and PYLY1. (**B**) The suppressor activities of PYLY2, PYLYDh, and PYLYCMa. (**C**) The suppressor activities of PYLY1 variants with the replacements of the identity elements. The cell suspensions of two or three *E. coli* clones transformed with the indicated variant genes were diluted successively (from top to bottom) and grown on the rich medium plates containing Cm at the indicated final concentrations with or without the supplementation of 3-iodotyrosine (IY).

**C.**


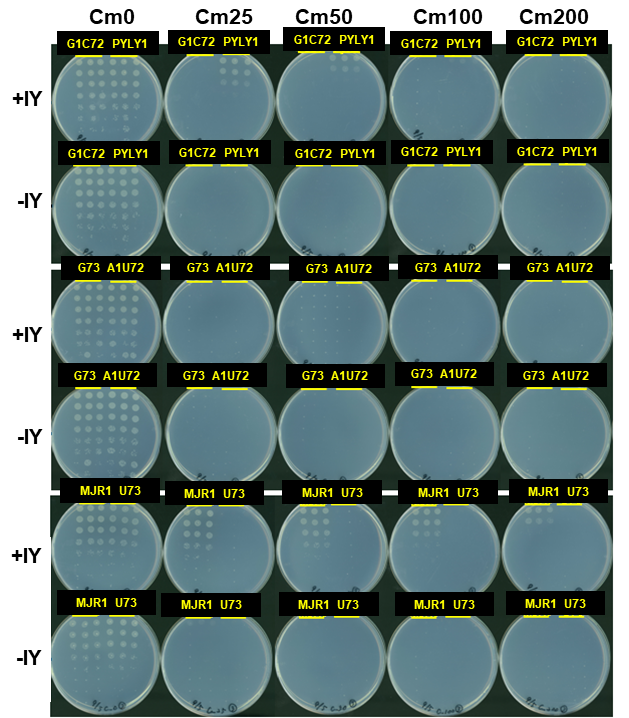


**Figure S1.** (continued)


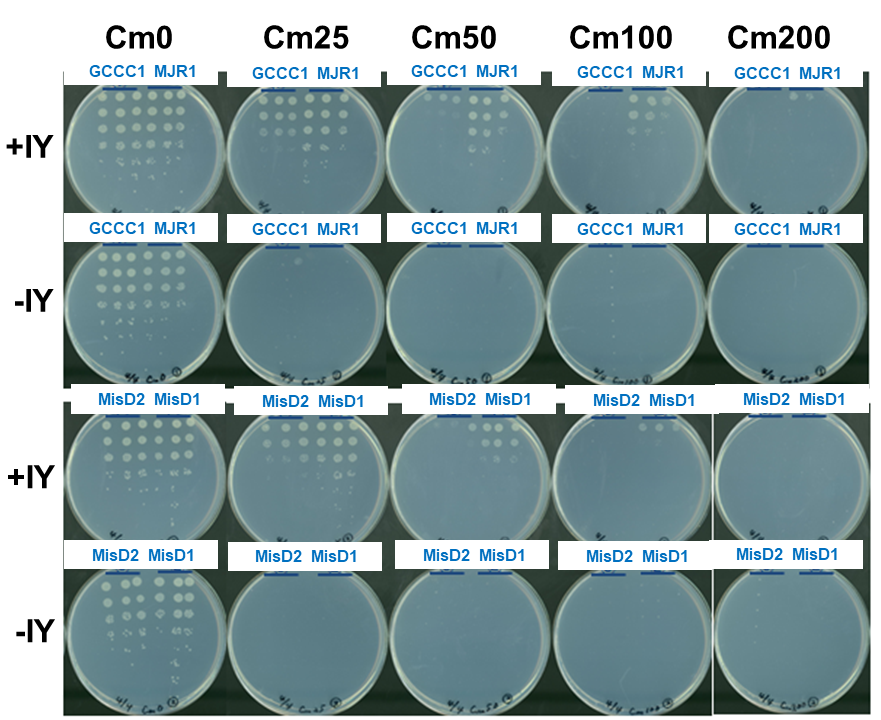


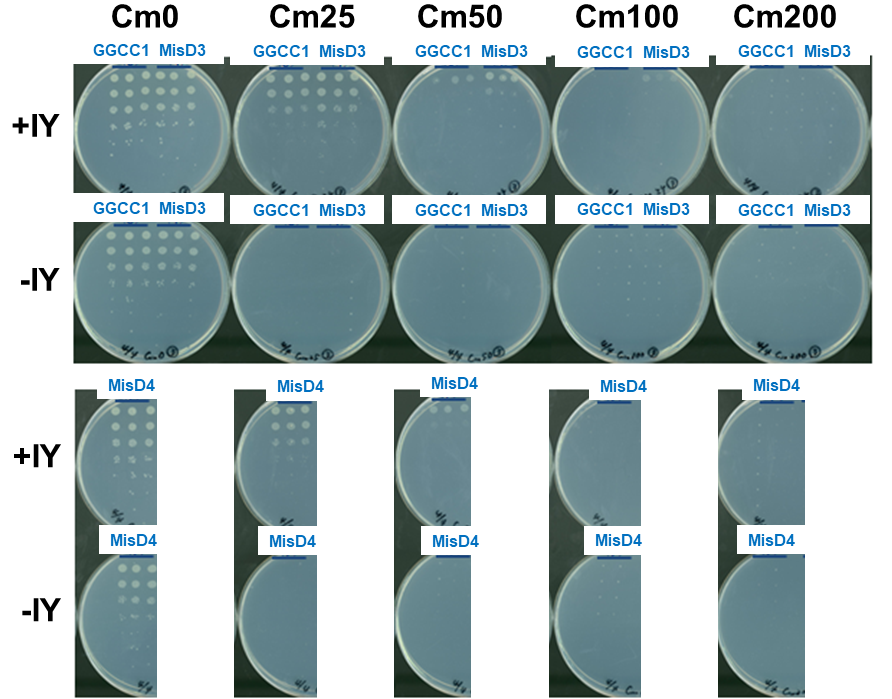


**Figure S2.** Amber suppressor activities of PYLY1 variants isolated from Selection 1. The cell suspensions of three *E. coli* clones transformed with the indicated variant genes were diluted successively (from top to bottom) and grown on the rich medium plates containing Cm at the indicated final concentrations with or without the supplementation of 3-iodotyrosine (IY). Irrelevant parts of the plates are removed from the figure.


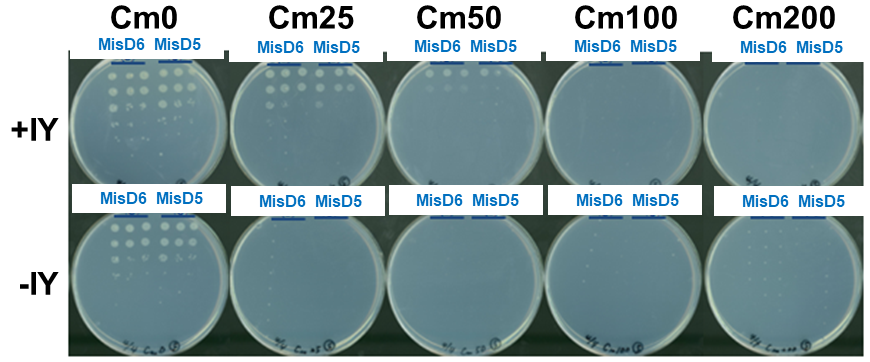


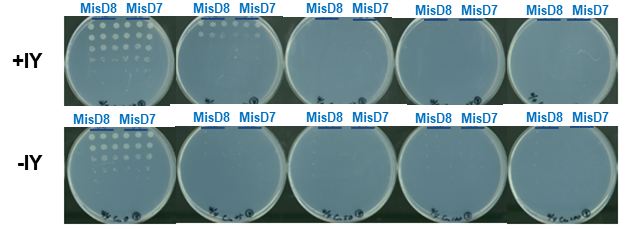


**Figure S2.** (continued)


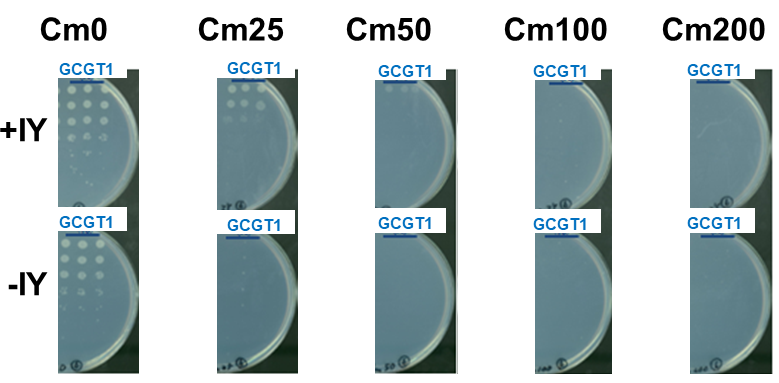


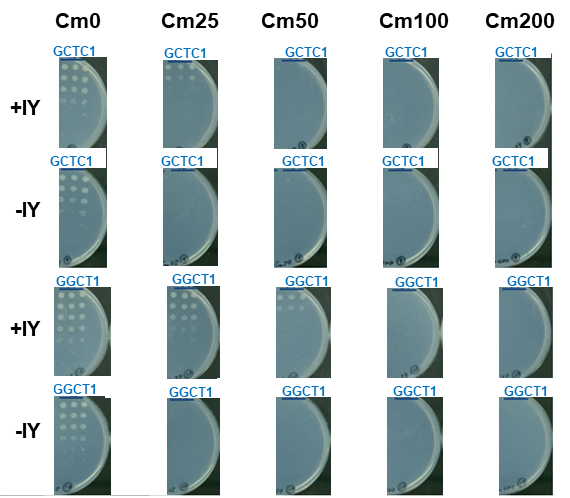


**Figure S2.** (concluded)

**A.**


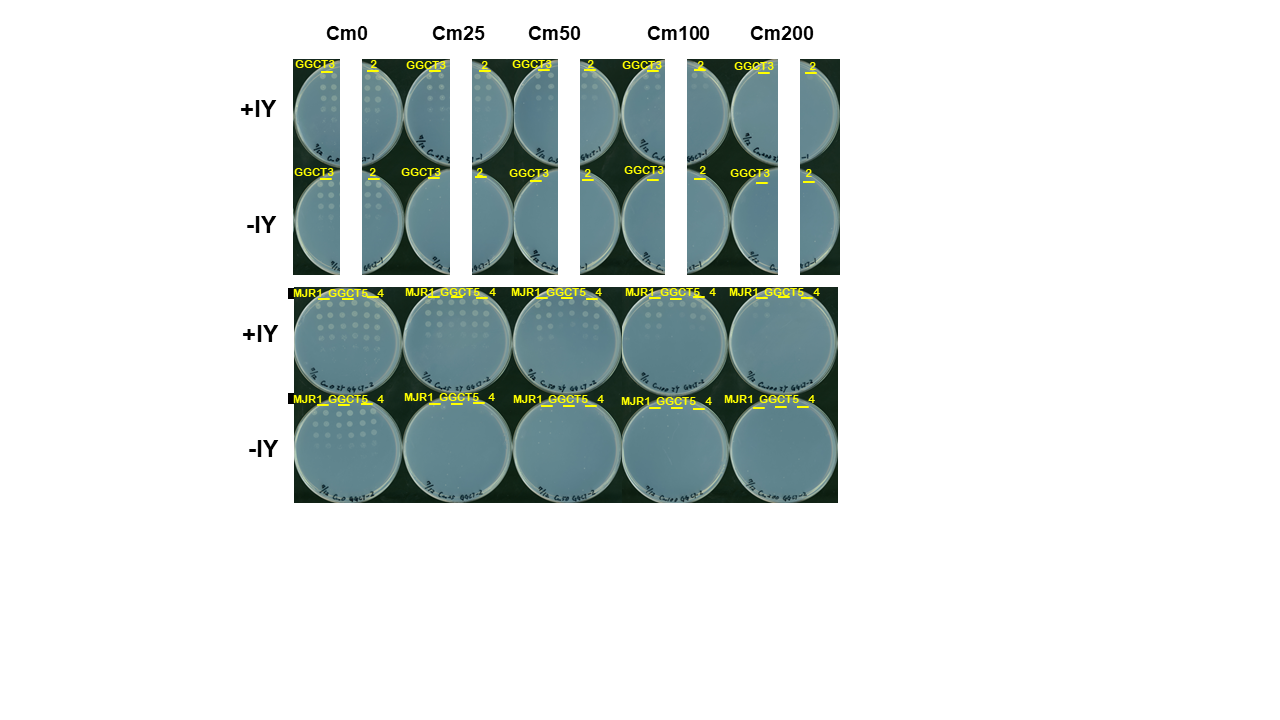


**B.**


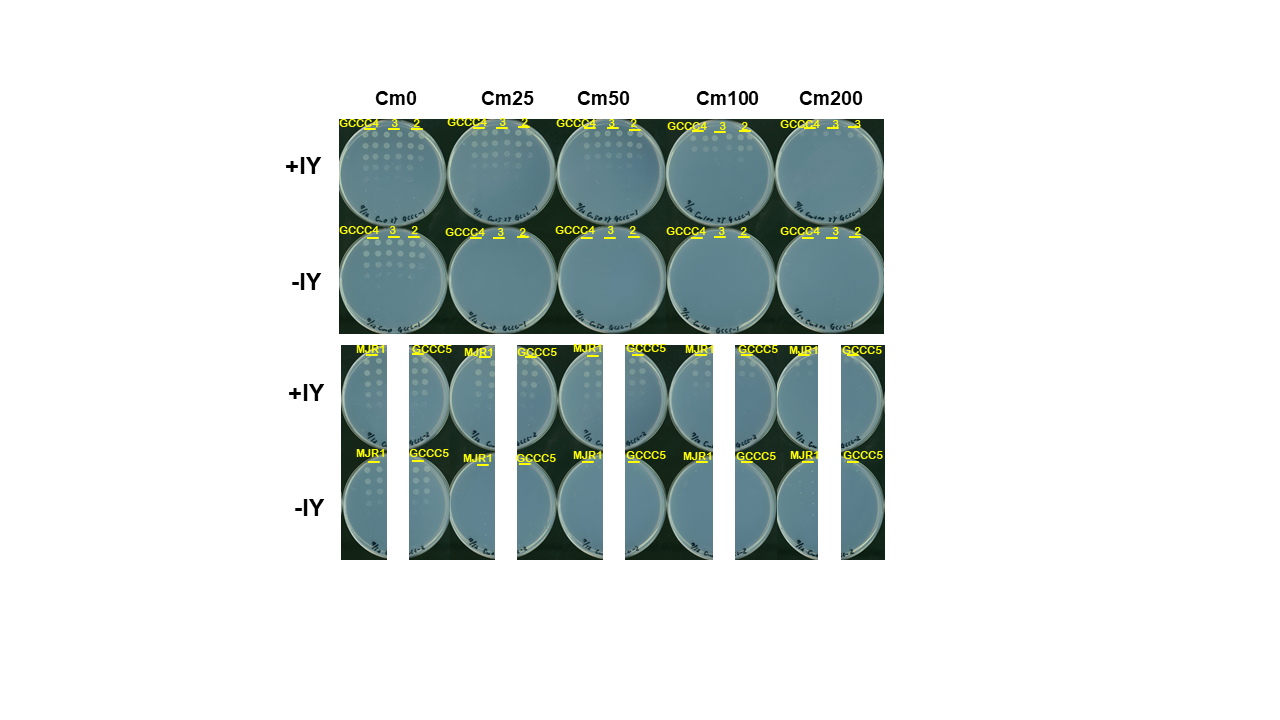


**Figure S3.** Amber suppressor activities of the variants of GGCT1 (**A**), GCCC (B), and PYLY1(GUUC) (**C**) isolated from Selection 2. The cell suspensions of two *E. coli* clones transformed with the indicated variant genes were diluted successively (from top to bottom) and grown on the rich medium plates containing Cm at the indicated final concentrations with or without the supplementation of 3-iodotyrosine (IY). Irrelevant parts of the plate are removed from the figure.

**C.**


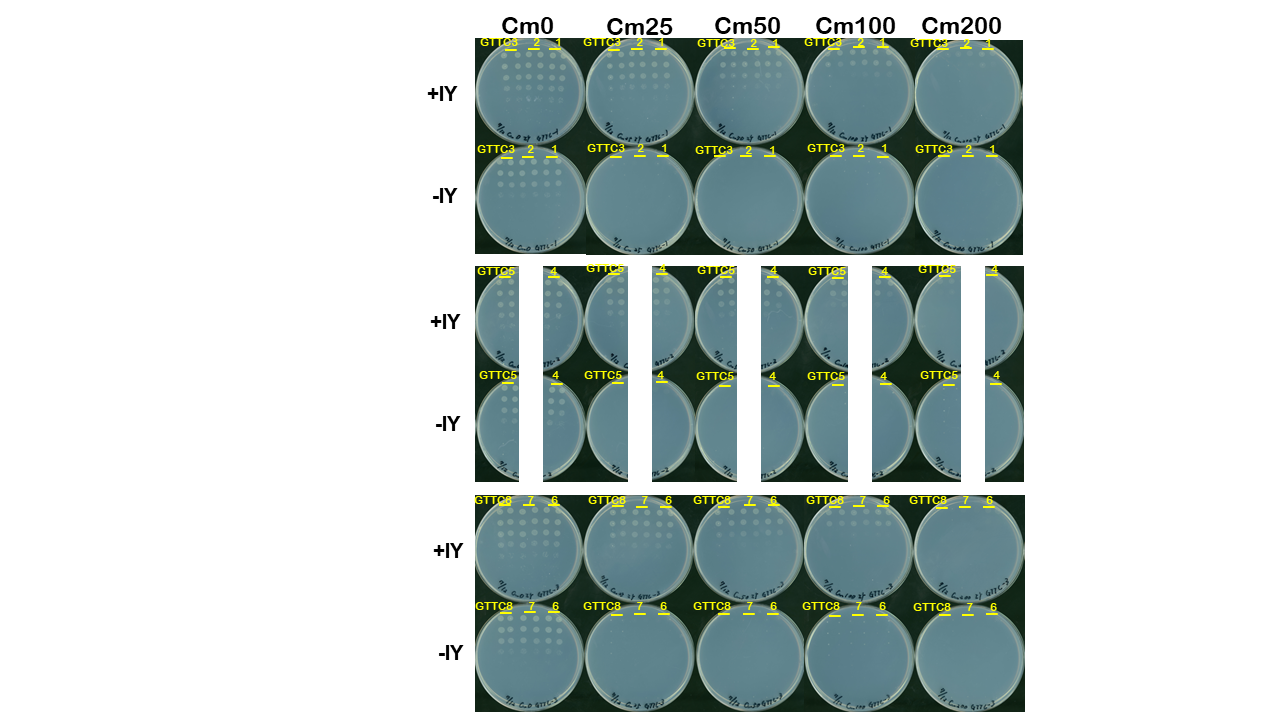


**Figure S3.** (continued)


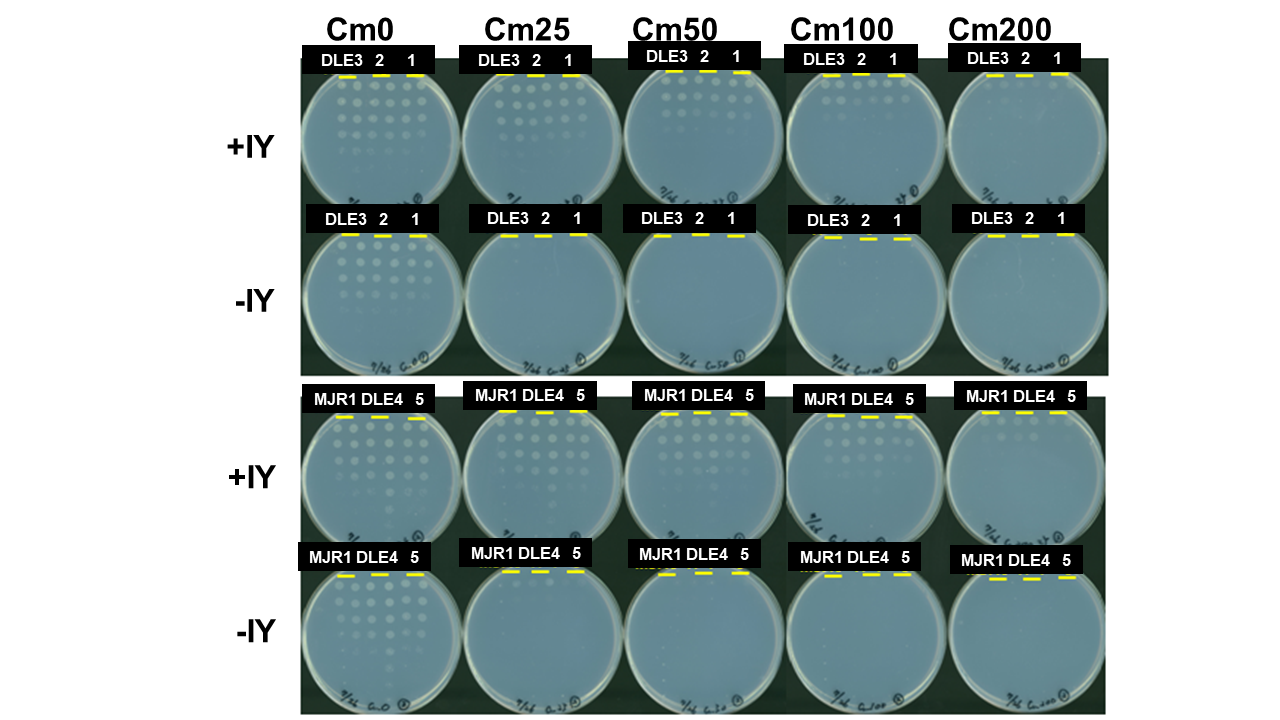


**Figure S4.** Amber suppressor activities of PYLY1 variants (DLE1—DLE5) from Selection 3. The cell suspensions of two *E. coli* clones transformed with the indicated variant genes were diluted successively (from top to bottom) and grown on the rich medium plates containing Cm at the indicated final concentrations with or without the supplementation of 3-iodotyrosine (IY).


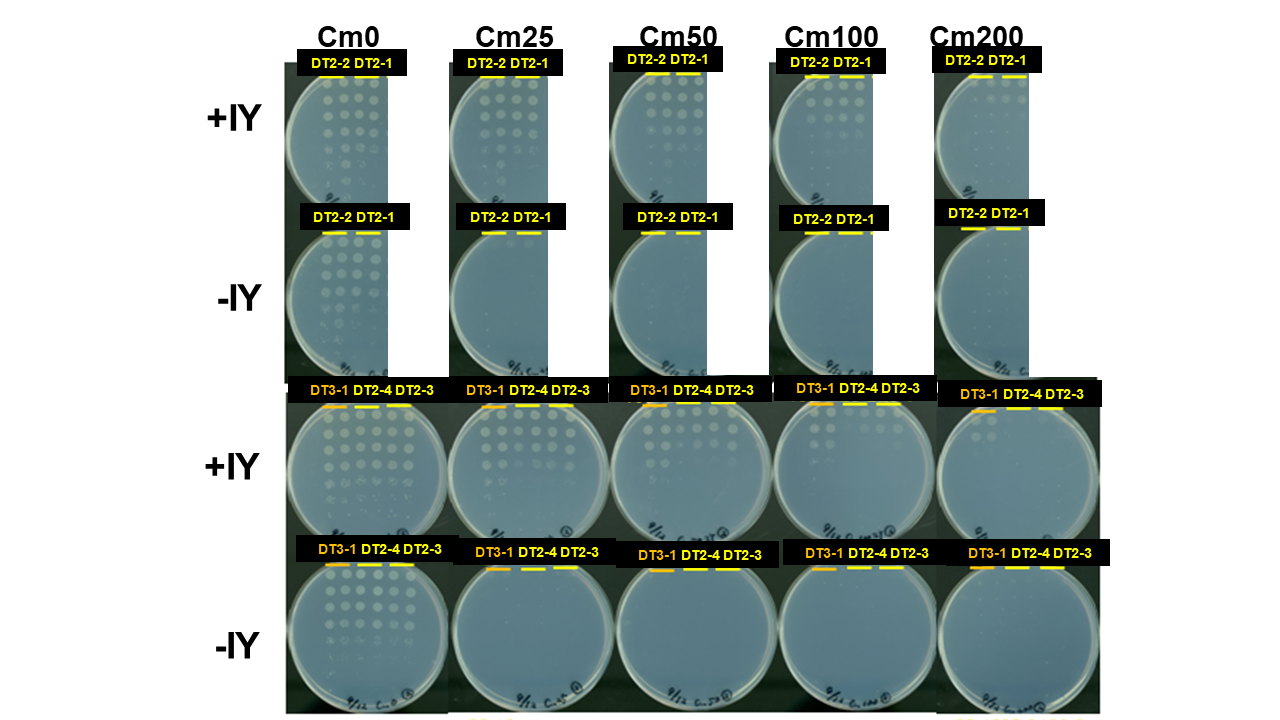


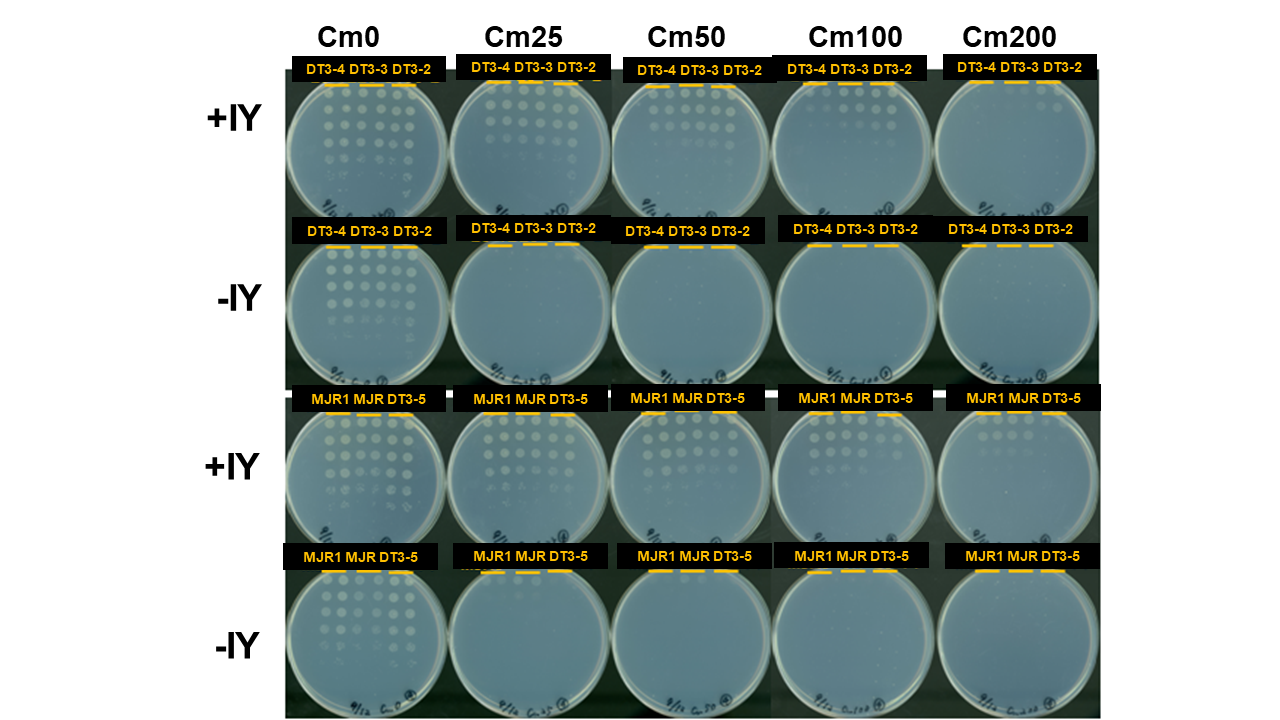


**Figure S5.** Amber suppressor activities of the PYLY1 and GGCT1 variants of the DT2- and DT3-series, respectively, isolated from Selection 4. The cell suspensions of two *E. coli* clones transformed with the indicated variant genes were diluted successively (from top to bottom) and grown on the rich medium plates containing Cm at the indicated final concentrations with or without the supplementation of 3-iodotyrosine (IY). Irrelevant parts of the plate are removed from the figure.


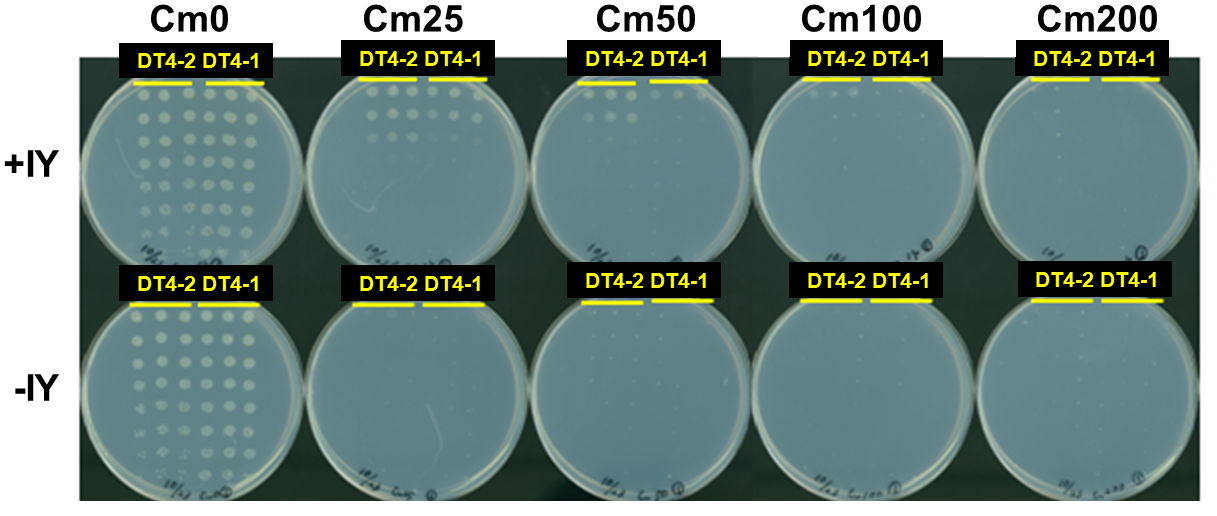


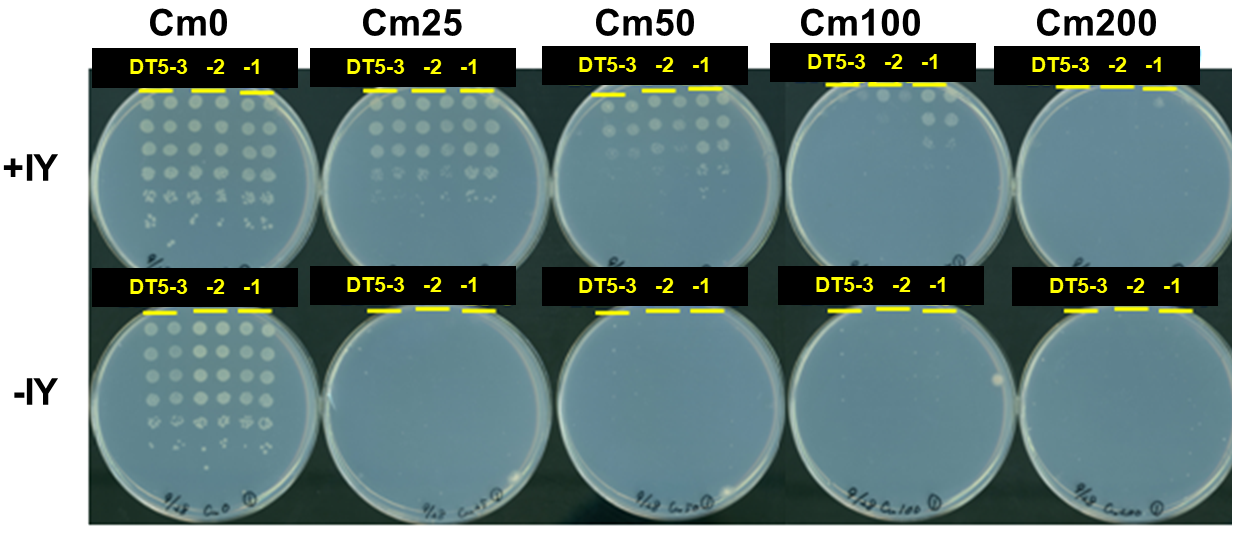


**Figure S6.** Amber suppressor activities of *Mj* tRNA^Tyr^ variants (DT4-, DT5-, and DT6-series) isolated from Selection 5. The cell suspensions of two or three *E. coli* clones transformed with the indicated variant genes were diluted successively (from top to bottom) and grown on the rich medium plates containing Cm at the indicated final concentrations with or without the supplementation of 3-iodotyrosine (IY).


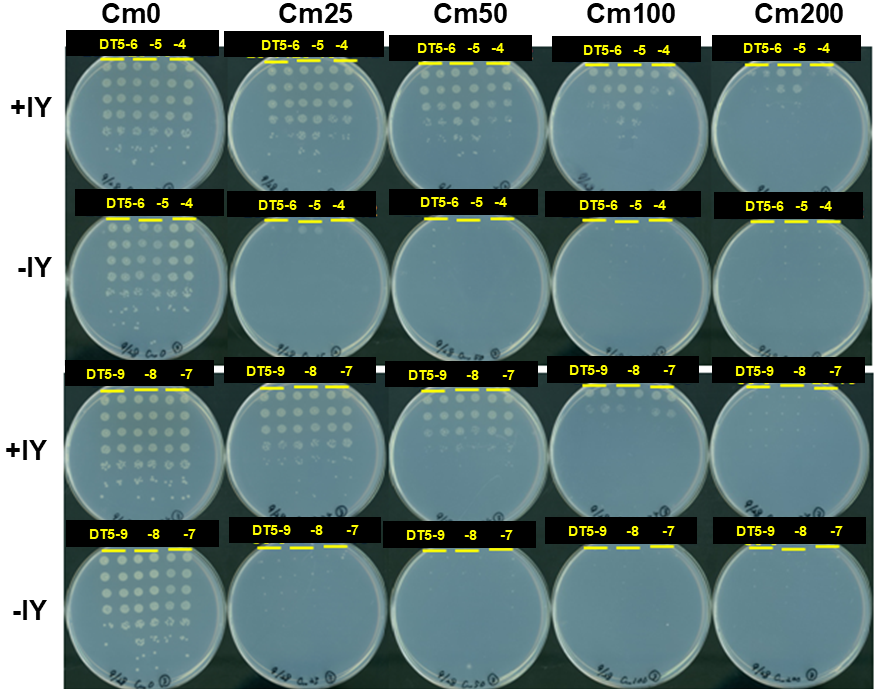


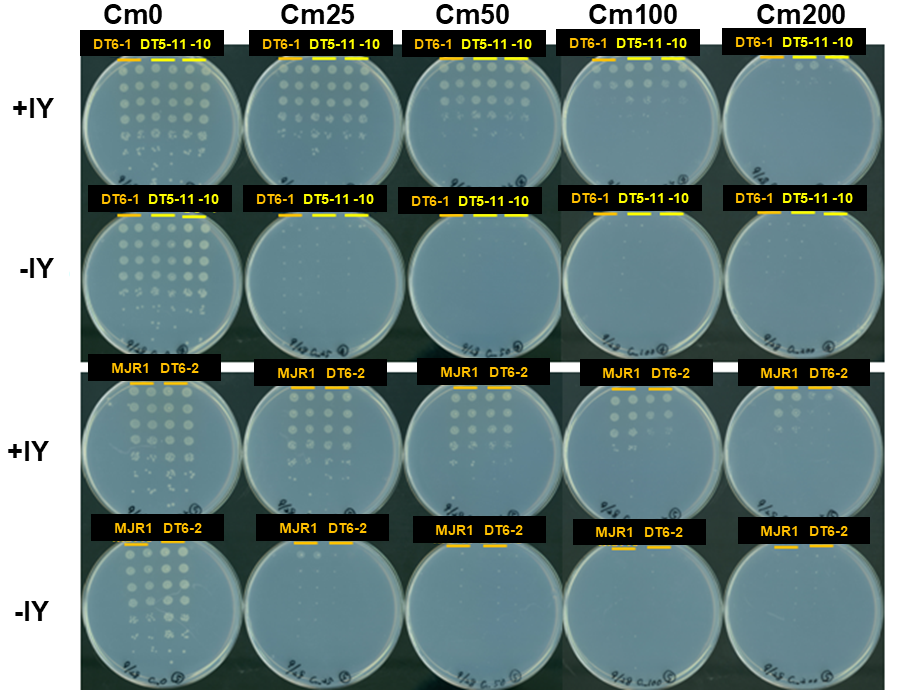


**Figure S6.** (continued)

**A.**


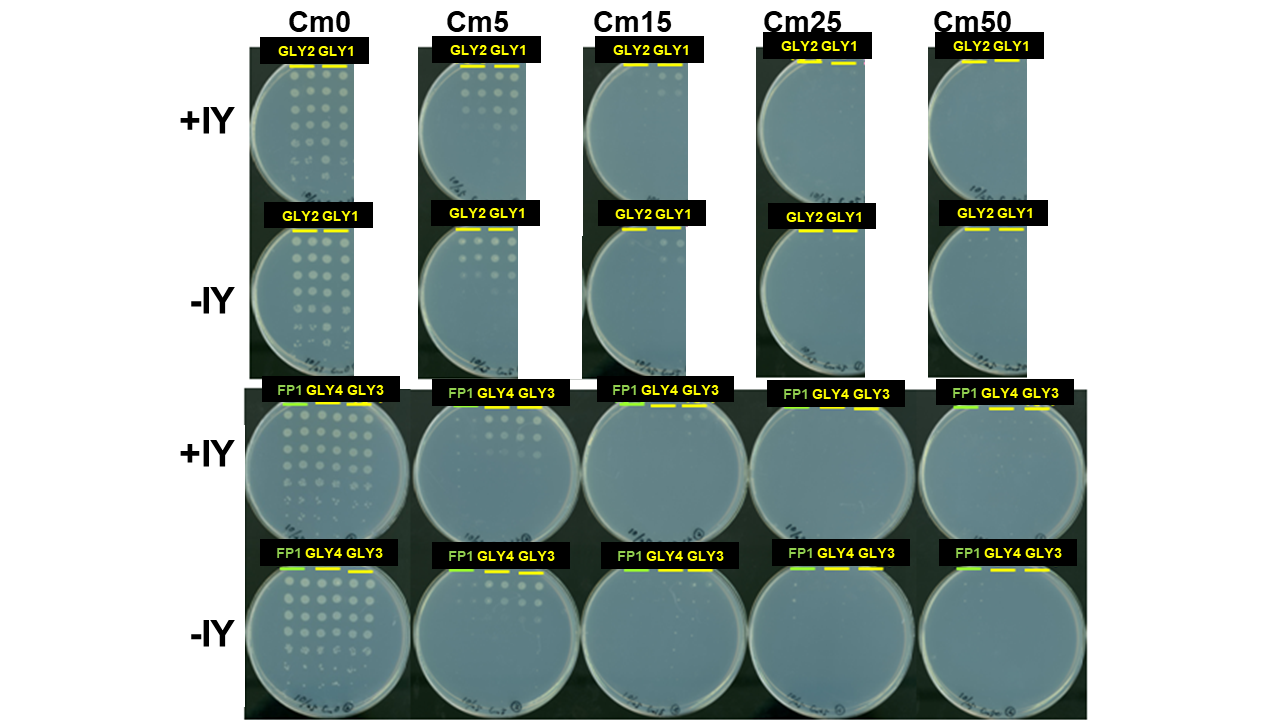


**Figure S7.** Amber suppressor activities of the GGCT1 isolated from Library 6a (**A**) and 6b (**B**) and the suppressor activity of GLNa (**C**). The cell suspensions of two *E. coli* clones transformed with the indicated variant genes were diluted successively (from top to bottom) and grown on the rich medium plates containing Cm at the indicated final concentrations with or without the supplementation of 3-iodotyrosine (IY). Irrelevant parts of the plate are removed from the figure.

**B.**


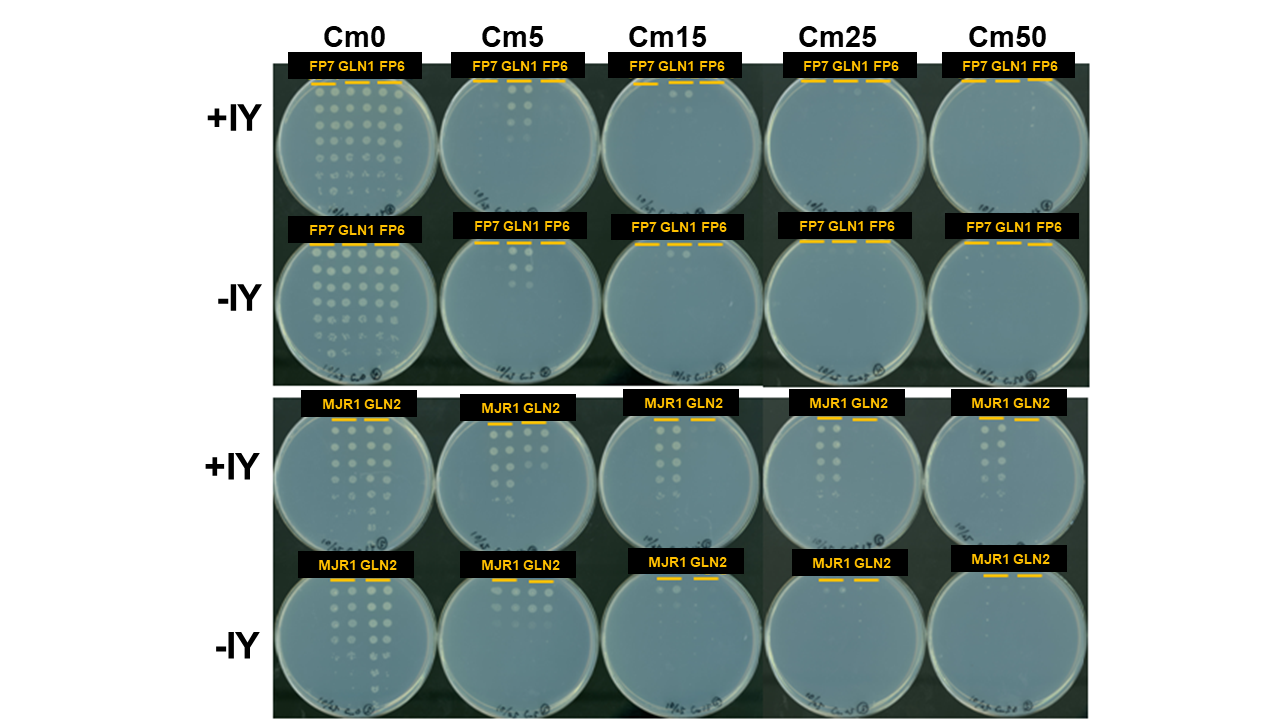


**C.**


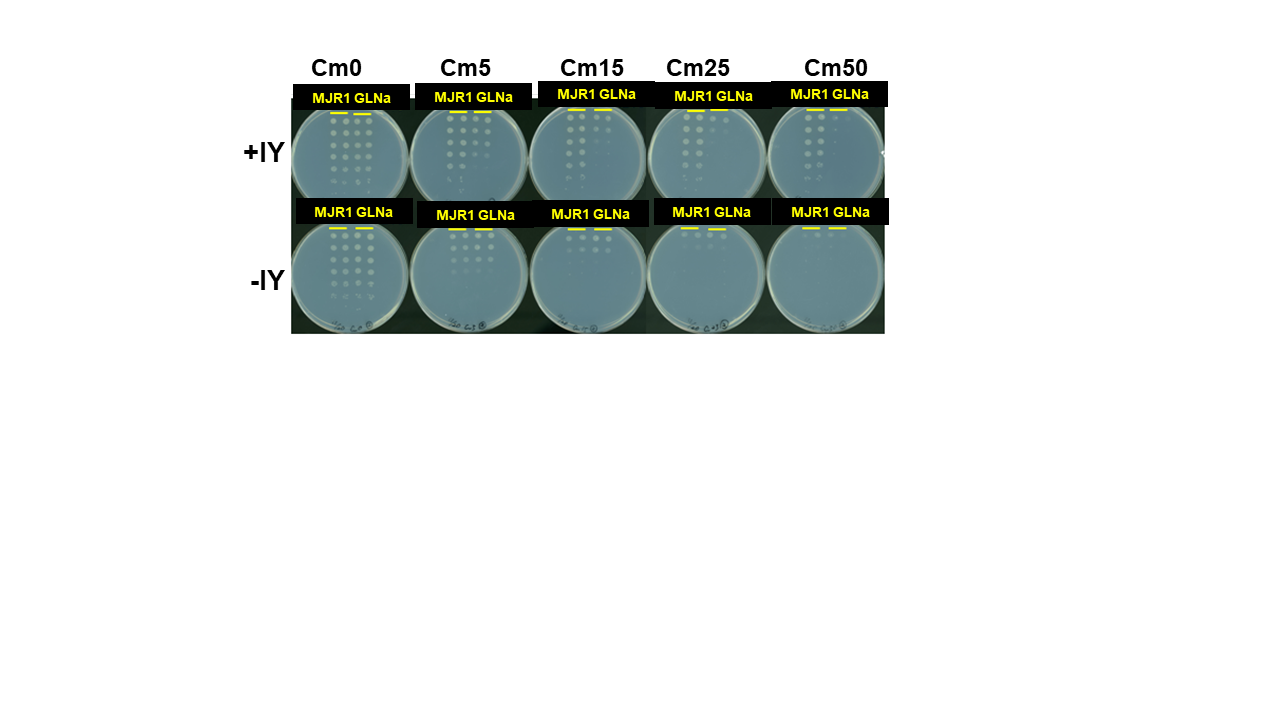


**Figure S7.** (continued)

GAATTCCGGATGAGCATTCATCAGGCGGGCAAGAATGTGAATAAAGGCCGGATAAAACTTGTGCTTATTTTTCTTTACGGTCTTTAAAAAGGCCGTAATATCCAGCTGAACGGTCTGGTTATAGGTACATTGAGCAACTGACTGAAATGCCTCAAAATGTTCTTTACGATGCCATTGGGATATATCAACCTAGGTATATCCCATGATTTTTTTCTCCATTTTAGCTTCCTTAGCTCCTGAAAATCTCGATAACTCAAAAAATACGCCCGGTAGTGATCTTATTTCATTATGGTGAAAGTTGGAACCTCTTACGTGCCGATCAACGTCTCATTTTCGCCAAAAGTTGGCCCAGGGCTTCCCGGTATCAACAGGGACACCAGGATTTATTTATTCTGCGAAGTGATCTTCCGTCACAGGTATTTATTCGGCGCAAAGTGCGTCGGGTGATGCTGCCAACTTACTGATTTAGTGTATGATGGTGTTTTTGAGGTGCTCCAGTGGCTTCTGTTTCTATCAGCTGTCCCTCCTGTTCAGCTACTGACGGGGTGGTGCGTAACGGCAAAAGCACCGCCGGACATCAGCGCTAGCGGAGTGTATACTGGCTTACTATGTTGGCACTGATGAGGGTGTCAGTGAAGTGCTTCATGTGGCAGGAGAAAAAAGGCTGCACCGGTGCGTCAGCAGAATATGTGATACAGGATATATTCCGCTTCCTCGCTCACTGACTCGCTACGCTCGGTCGTTCGACTGCGGCGAGCGGAAATGGCTTACGAACGGGGCGGAGATTTCCTGGAAGATGCCAGGAAGATACTTAACAGGGAAGTGAGAGGGCCGCGGCAAAGCCGTTTTTCCATAGGCTCCGCCCCCCTGACAAGCATCACGAAATCTGACGCTCAAATCAGTGGTGGCGAAACCCGACAGGACTATAAAGATACCAGGCGTTTCCCCCTGGCGGCTCCCTCGTGCGCTCTCCTGTTCCTGCCTTTCGGTTTACCGGTGTCATTCCGCTGTTATGGCCGCGTTTGTCTCATTCCACGCCTGACACTCAGTTCCGGGTAGGCAGTTCGCTCCAAGCTGGACTGTATGCACGAACCCCCCGTTCAGTCCGACCGCTGCGCCTTATCCGGTAACTATCGTCTTGAGTCCAACCCGGAAAGACATGCAAAAGCACCACTGGCAGCAGCCACTGGTAATTGATTTAGAGGAGTTAGTCTTGAAGTCATGCGCCGGTTAAGGCTAAACTGAAAGGACAAGTTTTGGTGACTGCGCTCCTCCAAGCCAGTTACCTCGGTTCAAAGAGTTGGTAGCTCAGAGAACCTTCGAAAAACCGCCCTGCAAGGCGGTTTTTTCGTTTTCAGAGCAAGAGATTACGCGCAGACCAAAACGATCTCAAGAAGATCATCTTATTAATCAGATAAAATATTTCTAGATTTCAGTGCAATTTATCTCTTCAAATGTAGCACCTGAAGTCAGCCCCATACGATATAAGTTGTAATTCTCATGTTTGACAGCTTATCATCGATAAGCTTGATGCGTGGAAGATTGATCGTCTTGCACCCTGAAAAGATGCAAAAATCTTGCTTTAATCGCTGGTACTCCTGATTCTGGCACTTTATTCTATGTCTCTTTCGCATCTGGCGAAAAGTCGTGTACCGGCAAAGGTGCAGTCGTTATATACTTGGAGATTCATatggacgaatttgaaatgataaagagaaacacatctgaaattatcagcgaggaagagttaagagaggttttaaaaaaagatgaaaaatctgcttacataggttttgaaccaagtggtaaaatacatttagggcattatctccaaataaaaaagatgattgatttacaaaatgctggatttgatataattatattgttggctgatttagccgcctatttaaaccagaaaggagagttggatgagattagaaaaataggagattataacaaaaaagtttttgaagcaatggggttaaaggcaaaatatgtttatggaagtgaattccagcttgataaggattatacactgaatgtctatagattggctttaaaaactaccttaaaaagagcaagaaggagtatggaacttatagcaagagaggatgaaaatccaaaggttgctgaagttatctatccaataatgcaggttaataccagtcattatttaggcgttgatgttgcagttggagggatggagcagagaaaaatacacatgttagcaagggagcttttaccaaaaaaggttgtttgtattcacaaccctgtcttaacgggtttggatggagaaggaaagatgagttcttcaaaagggaattttatagctgttgatgactctccagaagagattagggctaagataaagaaagcatactgcccagctggagttgttgaaggaaatccaataatggagatagctaaatacttccttgaatatcctttaaccataaaaaggccagaaaaatttggtggagatttgacagttaatagctatgaggagttagagagtttatttaaaaataaggaattgcatccaatgcgtttaaaaaatgctgtagctgaagaacttataaagattttagagccaattagaaagagattaTAAAGATCTGGATCCTCTACGCCGGACGCATCGTGGCCGGCATCACCGGCGCCACAGGTGCGGTTGCTGGCGCCTATATCGCCGACATCACCGATGGGGAAGATCGGGCTCGCCACTTCGGGCTCATGAGGCATGCGGCGCCGCTTCTTTGAGCGAACGATCAAAAATAAGTGGCGCCCCATCAAAAAAATATTCTCAACATAAAAAACTTTGTGTAATACTTGTAACGCTGCCATCAGATTGGAAATTTTTGATCCTTAGCGAAAGCTAAGGATTTTTTTTAGTCGACCGATGCCCTTGAGAGCCTTCAACCCAGTCAGCTCCTTCCGGTGGGCGCGGGGCATGACTATCGTCGCCGCACTTATGACTGTCTTCTTTATCATGCAACTCGTAGGACAGGTGCCGGCAGCGCTCTGGGTCATTTTCGGCGAGGACCGCTTTCGCTGGAGCGCGACGATGATCGGCCTGTCGCTTGCGGTATTCGGAATCTTGCACGCCCTCGCTCAAGCCTTCGTCACTGGTCCCGCCACCAAACGTTTCGGCGAGAAGCAGGCCATTATCGCCGGCATGGCGGCCGAGGCCATCCAGCCTCGCGTCGCGATAAAAAAAATCCTTAGCTTTCGCTAAGGATCAAAAATTTCCAAAGTGTTACAACCAATTAACCAATTCTGATTAGAAAAACTCATCGAGCATCAAATGAAACTGCAATTTATTCATATCAGGATTATCAATACCATATTTTTGAAAAAGCCGTTTCTGTAATGAAGGAGAAAACTCACCGAGGCAGTTCCATAGGATGGCAAGATCCTGGTATCGGTCTGCGATTCCGACTCGTCCAACATCAATACAACCTATTAATTTCCCCTCGTCAAAAATAAGGTTATCAAGTGAGAAATCACCATGAGTGACGACTGAATCCGGTGAGAATGGCAAAAGCTTATGCATTTCTTTCCAGACTTGTTCAACAGGCCAGCCATTACGCTCGTCATCAAAATCACTCGCATCAACCAAACCGTTATTCATTCGTGATTGCGCCTGAGCGAGACGAAATACGCGATCGCTGTTAAAAGGACAATTACAAACAGGAATCGAATGCAACCGGCGCAGGAACACTGCCAGCGCATCAACAATATTTTCACCTGAATCAGGATATTCTTCTAATACCTGGAATGCTGTTTTCCCGGGGATCGCAGTGGTGAGTAACCATGCATCATCAGGAGTACGGATAAAATGCTTGATGGTCGGAAGAGGCATAAATTCCGTCAGCCAGTTTAGTCTGACCATCTCATCTGTAACATCATTGGCAACGCTACCTTTGCCATGTTTCAGAAACAACTCTGGCGCATCGGGCTTCCCATACAATCGATAGATTGTCGCACCTGATTGCCCGACATTATCGCGAGCCCATTTATACCCATATAAATCAGCATCCATGTTGGAATTTAATCGCGGCTTCGAGCAAGACGTTTCCCGTTGAATATGGCTCATAACACCCCTTGTATTACTGTTTATGTAAGCAGACAGTTTTATTGTTCATGATGATATATTTTTATCTTGTGCAATGTAACATCAGAGATTTTGAGACACAACGTGGCTTTGTTGAATAAATCGAACTTTTGCTGAGTTGAAGGATCAGATCACGCATCTTCCCGACAACGCAGACCGTTCCGTGGCAAAGCAAAAGTTCAAAATCACCAACTGGTCCACCTACAACAAAGCTCTCATCAACCGTGGCTCCCTCACTTTCTGGCTGGATGATGGGGCGATTCAGGCCTGGTATGAGTCAGCAACACCTTCTTCACGAGGGACGACCGGGTCGAATTTGCTTTCGAATTTCTGCCATTCATCCGCTTATTATCACTTATTCAGGCGTAGCACCAGGCGTTTAAGGGCACCAATAACTGCCTTAAAAAAATTACGCCCCGCCCTGCCACTCATCGCAGTACTGTTGTAATTCATTAAGCATTCTGCCGACATGGAAGCCATCACAGACGGCATGATGAACCTGAATCGCCAGCGGCATCAGCACCTTGTCGCCTTGCGTATAATATTTGCCCATGGTGAAAACGGGGGCGAAGAAGTTGTCCATATTGGCCACGTTTAAATCAAAACTGGTGAAACTCACCCAGGGATTGGCTGAGACGAAAAACATATTCTCAATAAACCCTTTAGGGAAATAGGCCAGGTTTTCACCGTAACACGCCACATCTTGCGAATATATGTGTAGAAACTGCCGGAAATCGTCGTGGTATTCACTCCAGAGCGATGAAAACGTTTCAGTTTGCTCATGGAAAACGGTGTAACAAGGGTGAACACTATCCCATATCACCAGCTCACCGTCTTTCATTGCCATACG

**Figure S8.** The base sequence of pTYR2541 (4,964 bases). The CAT amber mutant gene in the reversed direction is indicated in dark blue, with the amber mutation shown in red. The gene encoding iodoTyrRS-*mj*(D286R) is indicated in lower cases, with the His70Ala, Asp158Thr, Ile159Ser, and Asp286Arg substitutions indicated in red. The *lpp* promoter and *rrnC* terminator are indicated in green and blue, respectively, with the intervening *Bst*XI site underlined. A sequence fragment from plasmid pHSG299 containing the kanamycin resistance gene with a promoter is highlighted in grey.
