# Supplementary material for: Synthetic Tyrosine tRNA Molecules with Noncanonical Secondary Structures
Source: Int J Mol Sci. 2018 Dec 26;20(1):92. doi: 10.3390/ijms20010092 (PMC6337575; doi:10.3390/ijms20010092)
Supplement: Supplementary file 1 [file ijms-20-00092-s001.zip › Supplementary materials/Supplemantary Tables S1 S2 revised.docx]

**Supplementary Materials**

Table S1

Table S2

**Table S1. Coding sequences for the tRNA molecules studied in the present study.**

The D, T, and anticodon stems are underlined. Bases in blue and black are derived from *M. jannaschii* tRNA^Tyr^ and tRNA^Pyl^, respectively, when they coexist in a sequence. Engineered positions are in red. Designed base substitutions and sequences are in green. “N” represents A, G, T, or C. The *in vivo* amber suppression activity of each tRNA in terms of the Cm resistance is shown at the right side of each sequence.

1. **Archaeal tRNA^Tyr^, tRNA^Pyl^, and tRNA variants in the tRNA^Pyl^ scaffold**

MJR: CCGGCGGTAGTTCAGCCTGGTAGAACGGCGGACTCTAAATCCGCATGTCGCTGGTTCAAATCCGGCCCGCCGGACCA Cm200

MJR1: CCGGCGGTAGTTCAGCAGGGCAGAACGGCGGACTCTAAATCCGCATGGCGCTGGTTCAAATCCGGCCCGCCGGACCA Cm200

*Mm* tRNA^Pyl^: GGAAACCTGATCATGTAGATCGAATGGACTCTAAATCCGTTCAGCCGGGTTAGATTCCCGGGGTTTCCGCCA N.D.

PYLY1: CCGGCGGTGATCATGTAGATCGAATGGACTCTAAATCCGTTCAGGCTGGTTAGATTCCGGCCCGCCGGACCA Cm100

PYLY2: CGAAACCTGATCATGTAGATCGAATGGACTCTAAATCCGTTCAGCCGGGTTAGATTCCCGGGGTTTCGACCA Cm100

PYLY1(G1C72): GCGGCGGTGATCATGTAGATCGAATGGACTCTAAATCCGTTCAGGCTGGTTAGATTCCGGCCCGCCGCACCA <Cm25

PYLY1(U1A72): TCGGCGGTGATCATGTAGATCGAATGGACTCTAAATCCGTTCAGGCTGGTTAGATTCCGGCCCGCCGAACCA <Cm25

PYLY1(G73): CCGGCGGTGATCATGTAGATCGAATGGACTCTAAATCCGTTCAGGCTGGTTAGATTCCGGCCCGCCGGGCCA <Cm25

PYLY1(U73): CCGGCGGTGATCATGTAGATCGAATGGACTCTAAATCCGTTCAGGCTGGTTAGATTCCGGCCCGCCGGTCCA <Cm25

PYLYDh: CGGGGGTGGATCGAATAGATCACACGGACTCTAAATTCGTGCAGGCGGGTGAAACTCCCGTACTCCCGACCA Cm50

PYLYCMa: CGGGGACGGTCCGGCGACCAGCGGGTCTCTAAAACCTGCCAGCGGGGTTCGACGCCCCGGTCTCTGACCA Cm200

1. **PYLY1 variants from Selection 1**

Library1: CCGGCGGNGNNNATGTANNNNNAATGGACTCTAAATCCGTTNNNGCTGGTTAGATTCCGGCCCGCCGGACCA

GCCC1: CCGGCGGGGCCCATGTAGGGCGAATGGACTCTAAATCCGTTAGAGCTGGTTAGATTCCGGCCCGCCGGACCA Cm25

GGCC1: CCGGCGGTGGCCATGTAGGCCAAATGGACTCTAAATCCGTTGTCGCTGGTTAGATTCCGGCCCGCCGGACCA Cm25

GCGT1: CCGGCGGAGCGTATGTAACGCCAATGGACTCTAAATCCGTTCTCGCTGGTTAGATTCCGGCCCGCCGGACCA Cm25

GCTC1: CCGGCGGTGCTCATGTAGAGCAAATGGACTCTAAATCCGTTTATGCTGGTTAGATTCCGGCCCGCCGGACCA Cm25

GGCT1: CCGGCGGTGGCTATGTAAGCCAAATGGACTCTAAATCCGTTCTCGCTGGTTAGATTCCGGCCCGCCGGACCA Cm50

MisD1: CCGGCGGAGATTATGTAATCGAAATGGACTCTAAATCCGTTGAAGCTGGTTAGATTCCGGCCCGCCGGACCA Cm50

MisD2: CCGGCGGTGGCGATGTAGCCAAAATGGACTCTAAATCCGTTCTCGCTGGTTAGATTCCGGCCCGCCGGACCA Cm25

MisD3: CCGGCGGCGCGTATGTACGCCGAATGGACTCTAAATCCGTTTGTGCTGGTTAGATTCCGGCCCGCCGGACCA Cm50

MisD4: CCGGCGGCGATTATGTAATCCCAATGGACTCTAAATCCGTTGGCGCTGGTTAGATTCCGGCCCGCCGGACCA Cm25

MisD5: CCGGCGGTGATTATGTAAATCGAATGGACTCTAAATCCGTTAAAGCTGGTTAGATTCCGGCCCGCCGGACCA Cm25

MisD6: CCGGCGGAGATTATGTAATCGAAATGGACTCTAAATCCGTTTTTGCTGGTTAGATTCCGGCCCGCCGGACCA Cm25

MisD7: CCGGCGGAGGGTATGTACTCGAAATGGACTCTAAATCCGTTCAAGCTGGTTAGATTCCGGCCCGCCGGACCA Cm25

MisD8: CCGGCGGAGAAAATGTATTCAAAATGGACTCTAAATCCGTTGAAGCTGGTTAGATTCCGGCCCGCCGGACCA Cm25

1. **GGCT1, GCCC1, and PYLY1(GUUC) variants from Selection 2**

Lib. 2a: CCGGCGGNGGCTATGTAAGCCNAATGGACTCTAAATCCGTTNNNGCTGGTTAGATTCCGGCCCGCCGGACCA

GGCT2: CCGGCGGCGGCTATGTAAGCC**A**AATGGACTCTAAATCCGTT**GTC**GCTGGTTAGATTCCGGCCCGCCGGACCA Cm100

GGCT3: CCGGCGGAGGCTATGTAAGCC**G**AATGGACTCTAAATCCGTT**GTT**GCTGGTTAGATTCCGGCCCGCCGGACCA Cm100

GGCT4: CCGGCGGAGGCTATGTAAGCC**A**AATGGACTCTAAATCCGTT**GTT**GCTGGTTAGATTCCGGCCCGCCGGACCA Cm100

GGCT5: CCGGCGGTGGCTATGTAAGCC**G**AATGGACTCTAAATCCGTT**GTC**GCTGGTTAGATTCCGGCCCGCCGGACCA Cm50

Lib. 2b: CCGGCGGNGCCCATGTAGGGCNAATGGACTCTAAATCCGTTNNNGCTGGTTAGATTCCGGCCCGCCGGACCA

GCCC2: CCGGCGG**T**GCCCATGTAGGGC**G**AATGGACTCTAAATCCGTT**TGA**GCTGGTTAGATTCCGGCCCGCCGGACCA Cm100

GCCC3: CCGGCGG**T**GCCCATGTAGGGC**A**AATGGACTCTAAATCCGTT**GGA**GCTGGTTAGATTCCGGCCCGCCGGACCA Cm100

GCCC4: CCGGCGG**T**GCCCATGTAGGGC**G**AATGGACTCTAAATCCGTT**TG**TGCTGGTTAGATTCCGGCCCGCCGGACCA Cm100

GCCC5: CCGGCGG**T**GCCCATGTAGGGC**G**AATGGACTCTAAATCCGTT**GGA**GCTGGTTAGATTCCGGCCCGCCGGACCA Cm100

Lib. 2c: CCGGCGGNGTTCATGTAGAACNAATGGACTCTAAATCCGTTNNNGCTGGTTAGATTCCGGCCCGCCGGACCA

GTTC1: CCGGCGG**T**GTTCATGTAGAAC**G**AATGGACTCTAAATCCGTT**TGA**GCTGGTTAGATTCCGGCCCGCCGGACCA Cm100

GTTC2: CCGGCGG**T**GTTCATGTAGAAC**A**AATGGACTCTAAATCCGTT**AGA**GCTGGTTAGATTCCGGCCCGCCGGACCA Cm100

GTTC3: CCGGCGG**T**GTTCATGTAGAAC**A**AATGGACTCTAAATCCGTTC**GA**GCTGGTTAGATTCCGGCCCGCCGGACCA Cm100

GTTC4: CCGGCGGAGTTCATGTAGAAC**G**AATGGACTCTAAATCCGTT**TGA**GCTGGTTAGATTCCGGCCCGCCGGACCA Cm100

GTTC5: CCGGCGG**T**GTTCATGTAGAAC**G**AATGGACTCTAAATCCGTT**AGA**GCTGGTTAGATTCCGGCCCGCCGGACCA Cm100

GTTC6: CCGGCGG**T**GTTCATGTAGAAC**G**AATGGACTCTAAATCCGTT**TG**CGCTGGTTAGATTCCGGCCCGCCGGACCA Cm100

GTTC7: CCGGCGG**T**GTTCATGTAGAAC**A**AATGGACTCTAAATCCGTT**TGA**GCTGGTTAGATTCCGGCCCGCCGGACCA Cm100

GTTC8: CCGGCGG**T**GTTCATGTAGAAC**G**AATGGACTCTAAATCCGTT**TG**TGCTGGTTAGATTCCGGCCCGCCGGACCA Cm100

1. **PYLY1 variants with randomized D loop sequences from Selection 3**

Library3: CCGGCGGNGATCNNNNNGATCNAATGGACTCTAAATCCGTTNNNGCTGGTTAGATTCCGGCCCGCCGGACCA

DLE1: CCGGCGGTGATCT**TGT**AGATCGAATGGACTCTAAATCCGTTTGAGCTGGTTAGATTCCGGCCCGCCGGACCA Cm100

DLE2: CCGGCGGAGATCA**TGT**TGATCAAATGGACTCTAAATCCGTTAGCGCTGGTTAGATTCCGGCCCGCCGGACCA Cm100

DLE3: CCGGCGGTGATCG**TGT**CGATCGAATGGACTCTAAATCCGTTAGAGCTGGTTAGATTCCGGCCCGCCGGACCA Cm100

DLE4: CCGGCGGAGATCC**TGT**GGATCGAATGGACTCTAAATCCGTTCTGGCTGGTTAGATTCCGGCCCGCCGGACCA Cm200

DLE5: CCGGCGGTGATCA**TGT**TGATCCAATGGACTCTAAATCCGTTTGCGCTGGTTAGATTCCGGCCCGCCGGACCA Cm100

1. **PYLY1 and GGCT1 variants with randomized D and T loop sequences from Selection 4**

Lib. 4a: CCGGCGGTGATCNNNNNGATCGAATGGACTCTAAATCCGTTCAGGCTGGTNNNANNCCGGCCCGCCGGACCA

DT2-1: CCGGCGGTGATCCT**GG**GGATCGAATGGACTCTAAATCCGTTCAGGCTGGT**TCG**ATTCCGGCCCGCCGGACCA Cm200

DT2-2: CCGGCGGTGATCAA**GG**TGATCGAATGGACTCTAAATCCGTTCAGGCTGGT**TCA**ATTCCGGCCCGCCGGACCA Cm100

DT2-3: CCGGCGGTGATCAT**GG**TGATCGAATGGACTCTAAATCCGTTCAGGCTGGT**TCA**ACTCCGGCCCGCCGGACCA Cm100

DT2-4: CCGGCGGTGATCAA**GG**AGATCGAATGGACTCTAAATCCGTTCAGGCTGGT**TCG**ATCCCGGCCCGCCGGACCA Cm50

Lib. 4b: CCGGCGGTGGCTNNNNNAGCCAAATGGACTCTAAATCCGTTCTCGCTGGTNNNANNCCGGCCCGCCGGACCA

DT3-1: CCGGCGGTGGCTCT**GG**GAGCCAAATGGACTCTAAATCCGTTCTCGCTGGT**TCG**ACTCCGGCCCGCCGGACCA Cm200

DT3-2: CCGGCGGTGGCTAC**GG**TAGCCAAATGGACTCTAAATCCGTTCTCGCTGGT**TCG**ACCCCGGCCCGCCGGACCA Cm200

DT3-3: CCGGCGGTGGCTCT**GG**TAGCCAAATGGACTCTAAATCCGTTCTCGCTGGT**TCG**ATTCCGGCCCGCCGGACCA Cm100

DT3-4: CCGGCGGTGGCTCG**GG**GAGCCAAATGGACTCTAAATCCGTTCTCGCTGGT**TCG**AGTCCGGCCCGCCGGACCA Cm50

DT3-5: CCGGCGGTGGCTTA**GG**GAGCCAAATGGACTCTAAATCCGTTCTCGCTGGT**TCG**ATTCCGGCCCGCCGGACCA Cm100

1. **Mj tRNATyr variants with randomized D loop sequences from Selection 5**

DT4 library:CCGGCGGNNGTTCNNNNNGAACGGCGGACTCTAAATCCGCNNNNGCTGGTTAGATTCCGGCCCGCCGGACCA

DT4-1: CCGGCGGAAGTTCAAGGTGAACGGCGGACTCTAAATCCGCAGGTGCTGGTTAGATTCCGGCCCGCCGGACCA Cm25

DT4-2: CCGGCGGTAGTTCGTACTGAACGGCGGACTCTAAATCCGCAGTTGCTGGTTAGATTCCGGCCCGCCGGACCA Cm50

DT5 library:CCGGCGGNNGTTCNNNNNNGAACGGCGGACTCTAAATCCGCNNNNGCTGGTTAGATTCCGGCCCGCCGGACCA

DT5-1: CCGGCGG**T**AGTTC**A**ACAA**A**GAACGGCGGACTCTAAATCCGCAAATGCTGGTTAGATTCCGGCCCGCCGGACCA Cm100

DT5-2: CCGGCGG**T**AGTTC**A**AAGGGGAACGGCGGACTCTAAATCCGCAAGTGCTGGTTAGATTCCGGCCCGCCGGACCA Cm50

DT5-3: CCGGCGG**T**GGTTC**A**AAGTCGAACGGCGGACTCTAAATCCGCAGGAGCTGGTTAGATTCCGGCCCGCCGGACCA Cm100

DT5-4: CCGGCGG**T**AGTTC**A**AACA**A**GAACGGCGGACTCTAAATCCGCTGGTGCTGGTTAGATTCCGGCCCGCCGGACCA Cm100

DT5-5: CCGGCGG**T**AGTTC**A**ACAT**A**GAACGGCGGACTCTAAATCCGCAGGTGCTGGTTAGATTCCGGCCCGCCGGACCA Cm200

DT5-6: CCGGCGG**T**AGTTC**A**TAGT**A**GAACGGCGGACTCTAAATCCGCAGGTGCTGGTTAGATTCCGGCCCGCCGGACCA Cm100

DT5-7: CCGGCGG**T**AGTTC**A**TGTC**A**GAACGGCGGACTCTAAATCCGCAAGAGCTGGTTAGATTCCGGCCCGCCGGACCA Cm100

DT5-8: CCGGCGGAAGTTC**A**ACGCGGAACGGCGGACTCTAAATCCGCAGGAGCTGGTTAGATTCCGGCCCGCCGGACCA Cm100

DT5-9: CCGGCGG**T**AGTTC**A**ATGG**A**GAACGGCGGACTCTAAATCCGCCTGTGCTGGTTAGATTCCGGCCCGCCGGACCA Cm100

DT5-10: CCGGCGG**T**GGTTC**A**GAGT**A**GAACGGCGGACTCTAAATCCGCAGATGCTGGTTAGATTCCGGCCCGCCGGACCA Cm100

DT5-11: CCGGCGG**T**GGTTC**A**ATTA**A**GAACGGCGGACTCTAAATCCGCAGGTGCTGGTTAGATTCCGGCCCGCCGGACCA Cm100

DT6 library:CCGGCGGNNGTTCNNNNNNNGAACGGCGGACTCTAAATCCGCNNNNGCTGGTTAGATTCCGGCCCGCCGGACCA

DT6-1: CCGGCGG**T**AGTTC**A**CTTGT**A**GAACGGCGGACTCTAAATCCGCAGGAGCTGGTTAGATTCCGGCCCGCCGGACCA Cm100

DT6-2: CCGGCGG**T**AGTTC**A**ACTTT**A**GAACGGCGGACTCTAAATCCGCGAATGCTGGTTAGATTCCGGCCCGCCGGACCA Cm100

1. **GGCT1 variants from Selection 6 and a designed variant for glutaminylation**

Lib. 6a: GCGGCGGTGGCTNNNNNAGCCAAATGGACTCTAAATCCGTTCTCGCTGGTNNNANNCCGGCCCGCCGCNCCA

GLY1: GCGGCGGTGGCTGT**GG**TAGCCAAATGGACTCTAAATCCGTTCTCGCTGGT**TCG**AGTCCGGCCCGCCGCTCCA Cm15*

GLY2: GCGGCGGTGGCTGG**GG**TAGCCAAATGGACTCTAAATCCGTTCTCGCTGGT**TCG**ATTCCGGCCCGCCGCTCCA Cm5*

GLY3: GCGGCGGTGGCTAT**GG**TAGCCAAATGGACTCTAAATCCGTTCTCGCTGGT**TCG**AGCCCGGCCCGCCGCTCCA Cm5*

GLY4: GCGGCGGTGGCTCA**GG**AAGCCAAATGGACTCTAAATCCGTTCTCGCTGGT**TCG**ATTCCGGCCCGCCGCTCCA Cm5*

FP1: GCGGCGGTGGCTTTGGAAGCCAAATGGACTCTAAATCCGTTCTCGCTGGTTGAAGTCCGGCCCGCCGCTCCA

FP2: GCGGCGGTGGCTGGTTCAGCCAAATGGACTCTAAATCCGTTCTCGCTGGTTTAACTCCGGCCCGCCGCTCCA

FP3: GCGGCGGTGGCTGATTAAGCCAAATGGACTCTAAATCCGTTCTCGCTGGTTAGATCCCGGCCCGCCGCACCA

FP4: GCGGCGGTGGCTCACATAGCCAAATGGACTCTAAATCCGTTCTCGCTGGTCCGAGTCCGGCCCGCCGCTCCA

Lib. 6b: DCGGCGGTGGCTNNNNNAGCCAAATGGACTCTAAATCCGTTCTCGCTGGTNNNANNCCGGCCCGCCGHNCCA

GLN1: TCGGCGGTGGCTAT**GG**TAGCCAAATGGACTCTAAATCCGTTCTCGCTGGT**TCG**ACTCCGGCCCGCCGCGCCA Cm15*

GLN2: TCGGCGGTGGCTCT**GG**GAGCCAAATGGACTCTAAATCCGTTCTCGCTGGT**TCG**ACTCCGGCCCGCCGTGCCA Cm5*

FP6: ACGGCGGTGGCTGTCCAAGCCAAATGGACTCTAAATCCGTTCTCGCTGGTGATAGTCCGGCCCGCCGAGCCA

FP7: TCGGCGGTGGCTGATTGAGCCAAATGGACTCTAAATCCGTTCTCGCTGGTATCATGCCGGCCCGCCGCACCA

GLNa: TGGGCGGTGGCTATGGTAGCCAAATGGACTCTAAATCCGTTCTCGCTGGTTCGACTCCGGCCCGCCCAGCCA Cm15-25*

Notes: “D” and “H” represent “A, G, or T” and “A, T, or C”, respectively. The *in vivo* suppressor activities indicated with asterisks were observed in the absence and presence of 3-iodotyrosine in the growth medium.

**Table S2. DNA oligomers for constructing tRNA genes**

“N”, “D”, and “H” represent “A, G, C, or T”, “A, G, or T”, and “A, T, or C”, respectively. Bases in italics are those outside the tRNA coding sequence. The sequences complementary with the *Bst*XI site of pTYR2541 are underlined.

**F-MJR:** *CGCATT*CCGGCGGTAGTTCAGCCTGGTAGAACGGCGGACTCTAAATCCGCATGTCGCTGGTTCAAATCCGGCCCGCCGGACCA*TTTATCACAGA*

**R-1:** *TGATAAA*TGGTCCGGCGGGCCGG, **R-2:** GGCTGAACTACCGCCGG*AATGCGTCTG*

**F-MJR1:** *CGCATT*CCGGCGGTAGTTCAGCAGGGCAGAACGGCGGACTCTAAATCCGCATGGCGCTGGTTCAAATCCGGCCCGCCGGACCA*TTTATCACAGA*

**R-1:** *TGATAAA*TGGTCCGGCGGGCCGG, **R-2:** TGCTGAACTACCGCCGG*AATGCGTCTG*

**F-*Mm* tRNA^Pyl^:**

*CGCATT*GGAAACCTGATCATGTAGATCGAATGGACTCTAAATCCGTTCAGCCGGGTTAGATTCCCGGGGTTTCCGCCA*TTTATCACAGA*

**R-1:** *TGATAAA*TGGCGGAAACCCCGGG, **R-2:** TACATGATCAGGTTTCC*AATGCGTCTG*

**F-PYRY1:** *CGCATT*CCGGCGGTGATCATGTAGATCGAATGGACTCTAAATCCGTTCAGGCTGGTTAGATTCCGGCCCGCCGGACCA*TTTATCACAGA*

**R-1:** *TGATAAA*TGGTCCGGCGGGCCGG, **R-2:** TACATGATCACCGCCGG*AATGCGTCTG*

**F-PYRY2:** *CGCATT*CGAAACCTGATCATGTAGATCGAATGGACTCTAAATCCGTTCAGCCGGGTTAGATTCCCGGGGTTTCGACCA*TTTATCACAGA*

**R-1:** *TGATAAA*TGGTCGAAACCCCGGG, **R-2:** TACATGATCAGGTTTCG*AATGCGTCTG*

**F-PYRY1(G1C72):** *CGCATT*GCGGCGGTGATCATGTAGATCGAATGGACTCTAAATCCGTTCAGGCTGGTTAGATTCCGGCCCGCCGCACCA*TTTATCACAGA*

**R-1:** *TGATAAA*TGGTGCGGCGGGCCGG, **R-2:** TACATGATCACCGCCGC*AATGCGTCTG*

**F-PYRY1(U1A72):** *CGCATT*TGGCGGTGATCATGTAGATCGAATGGACTCTAAATCCGTTCAGGCTGGTTAGATTCCGGCCCGCCGAACCA*TTTATCACAGA*

**R-1:** *TGATAAA*TGGTTCGGCGGGCCGG, **R-2:** TACATGATCACCGCCA*AATGCGTCTG*

**F-PYRY1(G73):**  *CGCATT*CCGGCGGTGATCATGTAGATCGAATGGACTCTAAATCCGTTCAGGCTGGTTAGATTCCGGCCCGCCGGGCCA*TTTATCACAGA*

**R-1:** *TGATAAA*TGGCCCGGCGGGCCGG, **R-2:** TACATGATCACCGCCGG*AATGCGTCTG*

**F-PYRY1(U73):**  *CGCATT*CCGGCGGTGATCATGTAGATCGAATGGACTCTAAATCCGTTCAGGCTGGTTAGATTCCGGCCCGCCGGTCCA*TTTATCACAGA*

**R-1:** *TGATAAA*TGGACCGGCGGGCCGG, **R-2:** TACATGATCACCGCCGG*AATGCGTCTG*

**F-PYRYDh:** *CGCATT*CGGGGGTGGATCGAATAGATCACACGGACTCTAAATTCGTGCAGGCGGGTGAAACTCCCGTACTCCCGACCA*TTTATCACAGA*

**R-1:** *TGATAAA*TGGTCGGGAGTACGGG, **R-2:** TATTCGATCCACCCCCG*AATGCGTCTG*

**F-PYRYCMa:** *CGCATT*CGGGGACGGTCCGGCGACCAGCGGGTCTCTAAAACCTGCCAGCGGGGTTCGACGCCCCGGTCTCTGACCA*TTTATCACAGA*

**R-1:** *TGATAAA*TGGTCAGAGACCGGGG, **R-2:** TCGCCGGACCGTCCCCG*AATGCGTCTG*

**F-Library 1:**

*CGCATT*CCGGCGGNGNNNATGTANNNNNAATGGACTCTAAATCCGTTNNNGCTGGTTAGATTCCGGCCCGCCGGACCA*TTTATCACAGA*

**R-1:** *TGATAAA*TGGTCCGGCGGGCCGG, **R-2:** TACATAAACACCGCCGG*AATGCG*TCTG

**F-Library 2a:** *CGCATT*CCGGCGGNGGCTATGTAAGCCNAATGGACTCTAAATCCGTTNNNGCTGGTTAGATTCCGGCCCGCCGGACCA*TTTATCACAGA*

**R-1:** *TGATAAA*TGGTCCGGCGGGCCGG, **R-2:** TACATAGCCNCCGCCGG*AATGCGTCTG*

**F-Library 2b:**

*CGCATT*CCGGCGGNGCCCATGTAGGGCNAATGGACTCTAAATCCGTTNNNGCTGGTTAGATTCCGGCCCGCCGGACCA*TTTATCACAGA*

**R-1:** *TGATAAA*TGGTCCGGCGGGCCGG, **R-2:** TACATGGGCNCCGCCGG*AATGCGTCTG*

**F-Library 2c:** *CGCATT*CCGGCGGNGTTCATGTAGAACNAATGGACTCTAAATCCGTTNNNGCTGGTTAGATTCCGGCCCGCCGGACCA*TTTATCACAGA*

**R-1:** *TGATAAA*TGGTCCGGCGGGCCGG, **R-2:** TACATGAACNCCGCCGG*AATGCGTCTG*

**F-Library 3:**

*CGCATT*CCGGCGGNGATCNNNNNGATCNAATGGACTCTAAATCCGTTNNNGCTGGTTAGATTCCGGCCCGCCGGACCA*TTTATCACAGA*

**R-1:** *TGATAAA*TGGTCCGGCGGGCCGG, **R-2:** NNNNNGATCNCCGCCGG*AATGCGTCTG*

**F-Library 4a:**

*CGCATTAGAATTT*CCGGCGGTGATCNNNNNGATCGAATGGACTCTAAATCCGTTCAGGCTGGTNNNANNCCGGCCCGCCGGACCA*TTTATCACAGA*

**R-1:** *TGATAAA*TGGTCCGGCGGGCCGG, **R-2:** CCGCCGG*AAATTCTAATGCGTCTG*

**F-Library 4b:** *CGCATTAGAATTT*CCGGCGGTGGCTNNNNNAGCCAAATGGACTCTAAATCCGTTCTCGCTGGTNNNANNCCGGCCCGCCGGACCA*TTTATCACAGA*

**R-1:** *TGATAAA*TGGTCCGGCGGGCCGG, **R-2:** CCGCCGG*AAATTCTAATGCGTCTG*

**F-DT4 library:** *CGCATTAGAATTT*CCGGCGGNNGTTCNNNNNGAACGGCGGACTCTAAATCCGCNNNNGCTGGTTAGATTCCGGCCCGCCGGACCA*TTTATCACAGA*

**R-1:** *TGATAAA*TGGTCCGGCGGGCCGG, **R-2:** CCGCCGG*AAATTCTAATGCGTCTG*

**F-DT5 library:** *CGCATTAGAATTT*CCGGCGGNNGTTCNNNNNNGAACGGCGGACTCTAAATCCGCNNNNGCTGGTTAGATTCCGGCCCGCCGGACCA*TTTATCACAGA*

**R-1:** *TGATAAA*TGGTCCGGCGGGCCGG, **R-2:** CCGCCGG*AAATTCTAATGCGTCTG*

**F-DT6 library:** *CGCATTAGAATTT*CCGGCGGNNGTTCNNNNNNNGAACGGCGGACTCTAAATCCGCNNNNGCTGGTTAGATTCCGGCCCGCCGGACCA*TTTATCACAGA*

**R-1:** *TGATAAA*TGGTCCGGCGGGCCGG, **R-2:** CCGCCGG*AAATTCTAATGCGTCTG*

**F-Library 6a:** *CGCATTAGAATTT*GCGGCGGTGGCTNNNNNAGCCAAATGGACTCTAAATCCGTTCTCGCTGGTNNNANNCCGGCCCGCCGCNCCA*TTTATCACAGA*

**R-1:** *TGATAAA*TGGNGCGGCGGGCCGG, **R-2:** CCGCCGC*AAATTCTAATGCGTCTG*

**F-Library 6b:** *CGCATTAGAATTT*DCGGCGGTGGCTNNNNNAGCCAAATGGACTCTAAATCCGTTCTCGCTGGTNNNANNCCGGCCCGCCGHNCCA*TTTATCACAGA*

**R-1:** *TGATAAA*TGGNDCGGCGGGCCGG, **R-2:** CCGCCGH*AAATTCTAATGCGTCTG*

**F-GLNa:**

*CGCATTAGAATTT*TGGGCGGTGGCTATGGTAGCCAAATGGACTCTAAATCCGTTCTCGCTGGTTCGACTCCGGCCCGCCCAGCCA*TTTATCACAGA*

**R-1:** *TGATAAA*TGGCTGGGCGGGCCGG, **R-2:** CCGCCCA*AAATTCTAATGCGTCTG*
